# Guillain–Barré syndrome and COVID-19 vaccination: a systematic review and meta-analysis

**DOI:** 10.1007/s00415-024-12186-7

**Published:** 2024-01-17

**Authors:** Stefano Censi, Giandomenico Bisaccia, Sabina Gallina, Valentina Tomassini, Antonino Uncini

**Affiliations:** 1 Department of Neuroscience, Imaging and Clinical Sciences, “G. d’Annunzio” University of Chieti-Pescara, Via Luigi Polacchi 11, 66100 Chieti, Italy; 2 Clinical Neurology, SS. Annunziata University Hospital, Chieti, Italy

**Keywords:** Guillain-Barré syndrome, Epidemiology, Meta-analysis, COVID-19 vaccines, Systematic review

## Abstract

**Background:**

Case-reports/series and cohorts of Guillain–Barré syndrome (GBS) associated with COVID-19 vaccination have been reported.

**Methods:**

A systematic review and meta-analysis of cohort studies of GBS after COVID-19 vaccination was carried out. Incidence and incidence rate ratio for a number of vaccine doses and risk of GBS, also considering the specific vaccine technology, were calculated in a random-effects model.

**Results:**

Of 554 citations retrieved, 518 were discarded as irrelevant. We finally included 15 studies. The random effect model yielded, regardless of the vaccine technology, 1.25 (95%CI 0.21; 2.83) GBS cases per million of COVID-19 vaccine doses, 3.93 (2.54; 5.54) cases per million doses for adenovirus-vectored vaccines and 0.69 (0.38; 1.06) cases per million doses for mRNA vaccines. The GBS risk was 2.6 times increased with the first dose. Regardless of the vaccine technology, the GBS risk was not increased but disaggregating the data it was 2.37 (1.67; 3.36) times increased for adenovirus-vectored vaccines and 0.32 (0.23; 0.47) for mRNA vaccines. Mortality for GBS after vaccination was 0.10 per million doses and 4.6 per GBS cases.

**Conclusions:**

Adenovirus-vectored vaccines showed a 2.4 times increased risk of GBS that was about seven times higher compared with mRNA-based vaccines. The decreased GBS risk associated with mRNA vaccines was possibly due to an elicited reduction of infections, including SARS-CoV-2, associated with GBS during the vaccination period. How adenovirus-vectored COVID-19 vaccines may trigger GBS is unclear and further studies should investigate the relationship between vaccine technologies and GBS risk.

**Supplementary Information:**

The online version contains supplementary material available at 10.1007/s00415-024-12186-7.

## Introduction

Guillain–Barré syndrome (GBS), a rare but potentially fatal disorder, is thought to be an autoimmune, post-infective polyradiculoneuropathy and is the most common cause of acute flaccid paralysis with an overall annual incidence of 1.1–1.8 cases per 100,000 [1, 2]. The GBS eponym is an umbrella term including a number of related autoimmune neuropathies including the GBS and Miller Fisher syndrome (MFS) variants with their subtypes [3]. In about two-thirds of patients, a gastrointestinal or respiratory infection precedes, within six weeks, the onset of GBS [1]. GBS has been also possibly associated with several vaccines and an excess of GBS cases was detected in the United States during the 1976/1977 “swine flu” (H1N1) vaccination campaign [4]. Epidemiological analyses showed that the rate of GBS attributable to the vaccine was approximately 4.9–5.9 per million vaccinations and greater from 14 to 28 days post-vaccination, with a 7.3-fold increase in the risk of developing GBS [4, 5]. Since then, several studies have assessed the risk of GBS following influenza vaccines with only two studies suggesting approximately one additional GBS case per one million vaccinations [6, 7]. An increased risk of GBS (3 excess cases per million of doses) has been also reported following the administration of the recombinant zoster vaccine [8]. No other vaccines have been convincingly linked to GBS [9].

Several vaccines against COVID-19 pathology, including the adenovirus-vectored Vaxzevria and the mRNA vaccine Comirnaty, have been approved for use in many countries since December 2020 and have been shown to reduce SARS-CoV-2 infections, viral transmission, patient hospitalizations and deaths in randomized controlled trials and real-world effectiveness studies [10–12]. However, the clinical trials were underpowered to detect rare adverse events such as GBS [11].

Soon after the start of large-scale vaccine programs, single cases and small series of GBS following vaccination with Vaxzevria were reported. In July 2021, both the FDA and the European Medicines Agency issued warnings of an increased GBS risk after adenovirus-vectored vaccines [13, 14]. In a UK-based cohort study, excess cases of GBS per one million people receiving Vaxzevria varied from 2.3 to 2.9 [15]. On May 2023 the WHO declared the end of the SARS-CoV-2 public health emergency of international with a total of 13.42 billion vaccine doses administered worldwide [16]. The aim of this meta-analysis is to reappraise, two years after the introduction of vaccines, the epidemiological data regarding the association between COVID-19 vaccination and GBS.

## Methods and materials

### Study design, search strategy and inclusion criteria

**Fig. 1:**
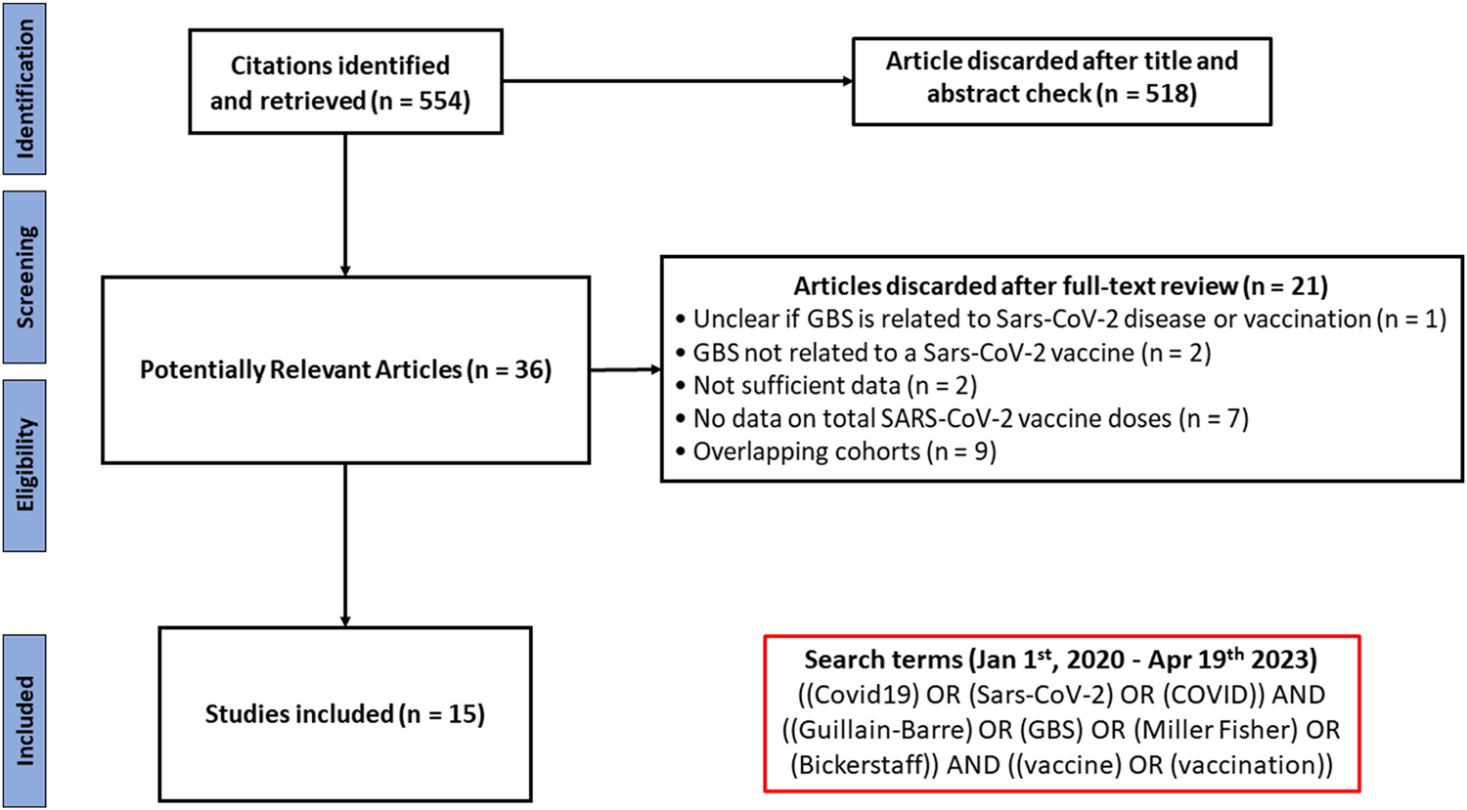
Flowchart of studies selection and search terms used

An author (SC) performed a systematic search on all studies published from 1 January 2020 to 19 April 2023 via PubMed-MEDLINE. The search terms used are reported in Fig. 1. All available peer-reviewed papers published in English, French, Italian, and Spanish were included. Letters, and commentaries that reported original data were also included. The reference lists of all relevant articles were also examined. No restriction on population gender, ethnicity, age, and medical history was applied. For missing or unclear information, we obtained further data consulting the original authors. List of included vaccines is available in the supplementary materials (SM) (Table S3). When more cohorts from the same area were present, we included the one with the more numerous population sample for each type of vaccine.

The inclusion criteria were: COVID-19 vaccination; diagnosis of GBS, MFS and their subtypes according to a clinical classification [3]; occurrence of GBS within 42 days after vaccination [17]; hospitalized patients. Diagnostic criteria adopted in each included study are summarized in SM.

### Quality assessment and publication bias

This study was registered in the International Prospective Register of Ongoing Systematic Reviews (PROSPERO) in 2023 (CRD42023433398) and was conducted complying with the Preferred Reporting Items for Systematic Reviews and Meta-Analyses guidelines [18]. Eligible cohort studies were assessed for quality and risk of bias through the Newcastle–Ottawa Scale [19].

### Statistical analysis

All analyses were performed using Microsoft Office and R software version 4.1.0. Packages utilised are detailed in the Supplementary Material [20].

For the study of GBS incidence following vaccination we adopted both Freeman-Tukey Transformation (FTT) and General Linear Mixed Model (GLMM). Average effects for the outcomes and 95% confidence interval (CI) were obtained using a random–effect model. We adopted both methods since conflicting opinions persist on which is the optimal method for meta-analysis of rare events [21]. We adopted the Mantel–Haenszel (MH) method in a random-effects model for calculating the Risk Ratio (RR) between observed and expected GBS cases after vaccination. Proportion of total variability due to between-study heterogeneity was estimated by Cochrane *Q* Chi^2^ statistics and *I*^2^ statistics [22]. Since our analysis dealt with rare events, we did not set a cut-off for homogeneity for Cochrane *Q* Chi^2^ test *p*-value and/or for *I*^2^ statistics. *I*^2^ represents what proportion of the observed variance is attributed to the variance in true effects rather than to sampling error. For a qualitative interpretation, *I*^2^ values lower than 30% were considered to represent low variability due to between-study heterogeneity, while values higher than 75% indicated considerably high variability [20]. The Knapp–Hartung adjustment was applied to the calculation of confidence intervals when more than five studies were available, while Paule–Mandel estimator was used to calculate tau^2^. Prediction intervals were based on t-distribution [22] and were calculated when more than six studies were available, acknowledging that the statistical accuracy of prediction intervals is inflated when dealing with rare events [23]. We built forest plots for each meta-analysis endpoint and then assessed for the presence of small-study effects and possible publication bias using Egger’s and Peters’ method for assessment of funnel plot asymmetry when more than 10 studies were available [24]. Wherever appropriate, we conducted an influence analysis and plotted the results. Outlier analysis was also implemented (studies with low standard error that still deviate substantially from the pooled effect are classified as outliers) [20].

For incidence analyses, we reported only the FTT outcomes in the main text; GLMM results and more detailed presentation of the meta-analyses is reported in "Methods and materials" section of in the supplementary information (SI).

## Results

Figure 1 is the flowchart of the systematic review. A total of 554 references were identified, of which 518, after reviewing the title and the abstract, were discarded as not pertinent.

Of 36 potentially relevant papers, we excluded 21 papers for the following reasons: uncertainty whether GBS was related to COVID-19 disease or vaccination (*n* = 1), GBS not related to a vaccine (*n* = 2), not sufficient data (*n* = 2), missing data on total COVID-19 vaccine doses (*n* = 7), possible overlapping patients (*n* = 9).

Overall, 15 studies were included [25–39]. The meta-analysis only deals with adeno-vectored and mRNA vaccines since very few data are available for other vaccine technologies.

### GBS incidence per COVID-19 vaccine doses administered regardless of the vaccine technology

**Fig. 2:**
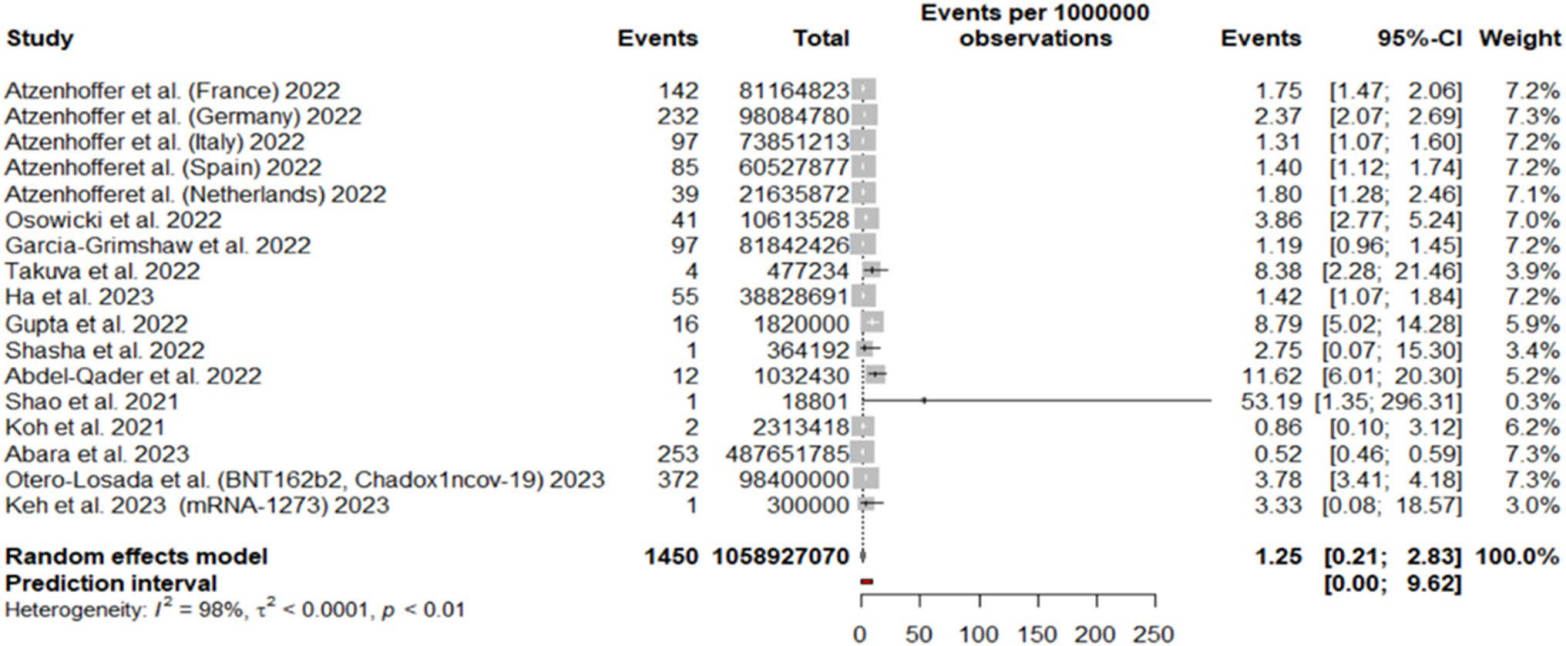
Forest plot of GBS incidence with respect to COVID-19 vaccine doses administered regardless of the vaccine technology. Events: number of GBS cases; Total: number of vaccine doses

We included 17 cohorts from 13 studies, collecting 1450 GBS cases over a total of 1,058,927,070 administered vaccine doses. The random-effects model yielded 1.25 GBS cases per million vaccine doses (95%CI 0.21; 2.83) (Fig. 2). The prediction interval yielded a range of 0 to 9.6 cases per million vaccine doses. Regional subgroup analyses showed for Asian countries 1.53 GBS cases per million vaccine doses (95%CI 0.00; 11.08), and for European countries 1.81 GBS cases per million vaccine doses (95%CI 1.08; 2.71) (SI).

### Risk ratio after first and second vaccination dose

**Fig. 3:**
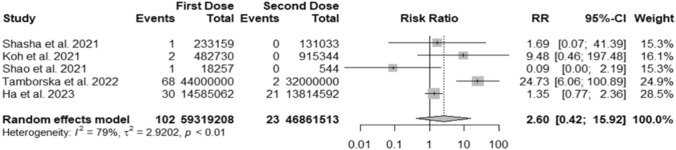
Forest plot about risk ratio between GBS cases linked to first and second doses of COVID-19 vaccine. Events: number of GBS cases; Total: number vaccine doses

We included five cohorts in the analysis of risk between GBS cases after the first and second doses of COVID-19 vaccination. We excluded vaccines that required only one vaccine dose (e.g., Jcovden). The MH method yielded an RR of 2.60 (95%CI 0.42, 15.92) for the first dose (Fig. 3).

### GBS incidence per COVID-19 vaccine technology

**Fig. 4:**
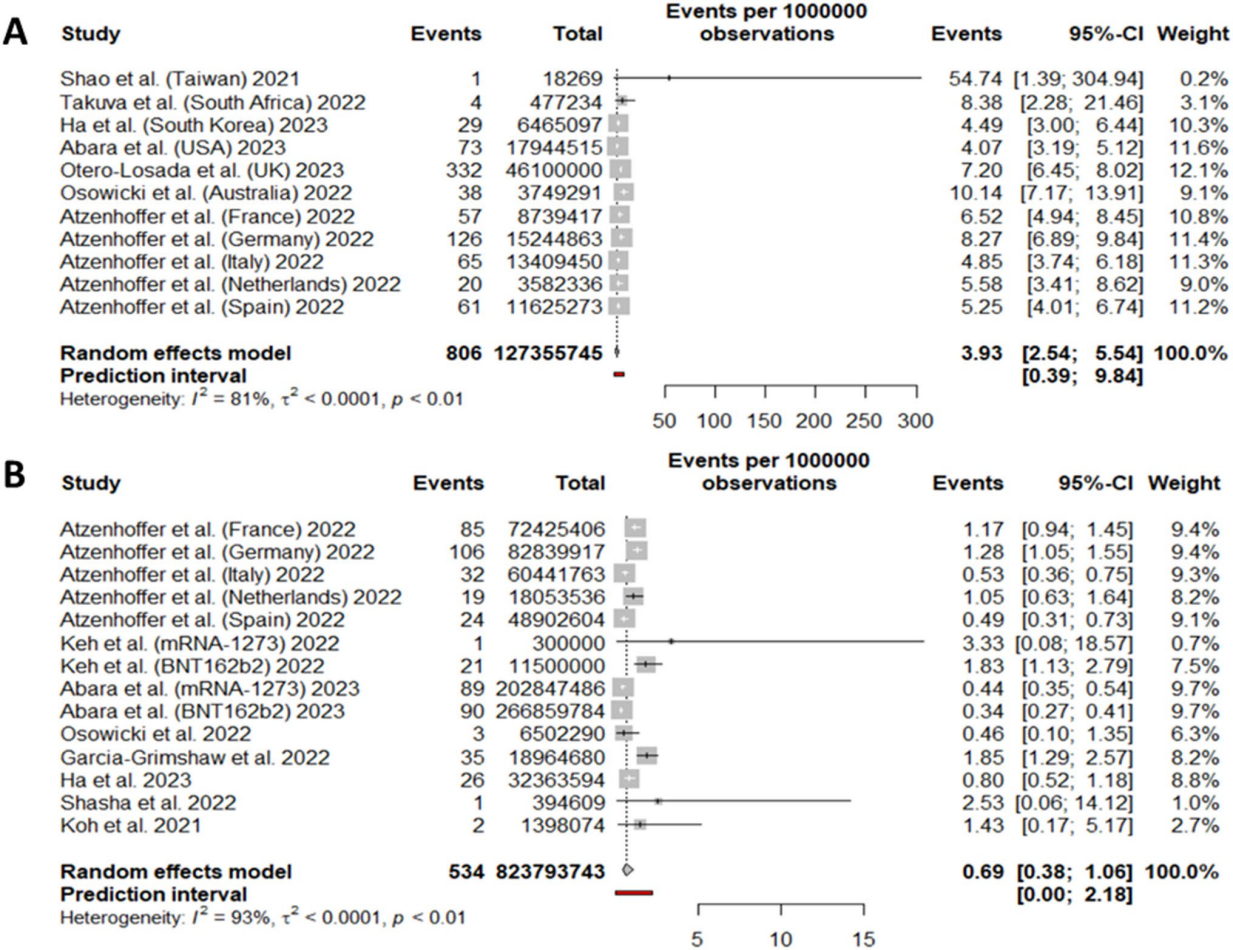
Forest plot of GBS incidence with respect to **A** adenoviral-vectored COVID-19 vaccine doses administered, and **B** with respect to mRNA-based COVID-19 vaccine doses administered. Events: number of GBS cases; Total: number of **A** adenoviral-vectored or **B** mRNA-based vaccine doses

For adenoviral-vectored vaccines, we included 11 cohorts from 7 studies, collecting 806 GBS cases over a total of 127,355,745 vaccine doses. The random-effect model yielded 3.93 (95%CI 2.54; 5.54) GBS cases per million doses of adenovirus-based COVID-19 vaccine (Fig. 4A). More specifically, for Vaxzevria the random-effect model proportion was 2.23 GBS cases every million doses (95%CI 0.00, 7.88). For Jcovden the random-effect model proportion was 6.63 for every million doses (95%CI 4.75, 8.80) (SI, Section 2).

For mRNA vaccines, we included 14 cohorts from 8 studies, collecting 534 GBS cases over a total of 823,793,743 vaccine doses. The random-effect model proportion yielded 0.69 (95%CI 0.38; 1.06) GBS cases per million doses of mRNA-based COVID-19 vaccine with a prediction interval from 0.00 to 2.18 (Fig. 4B).

Specifically, for Comirnaty the random-effect model proportion was 0.64 GBS cases every million doses (95%CI 0.12, 1.45), and for Spikevax the random-effect model proportion was 0.66 every million doses (95%CI 0.27, 1.21) with a prediction interval between 0.0 and 2.6 (SI, Section 2).

### Risk ratio between observed and expected GBS cases after COVID-19 vaccination

**Fig. 5:**
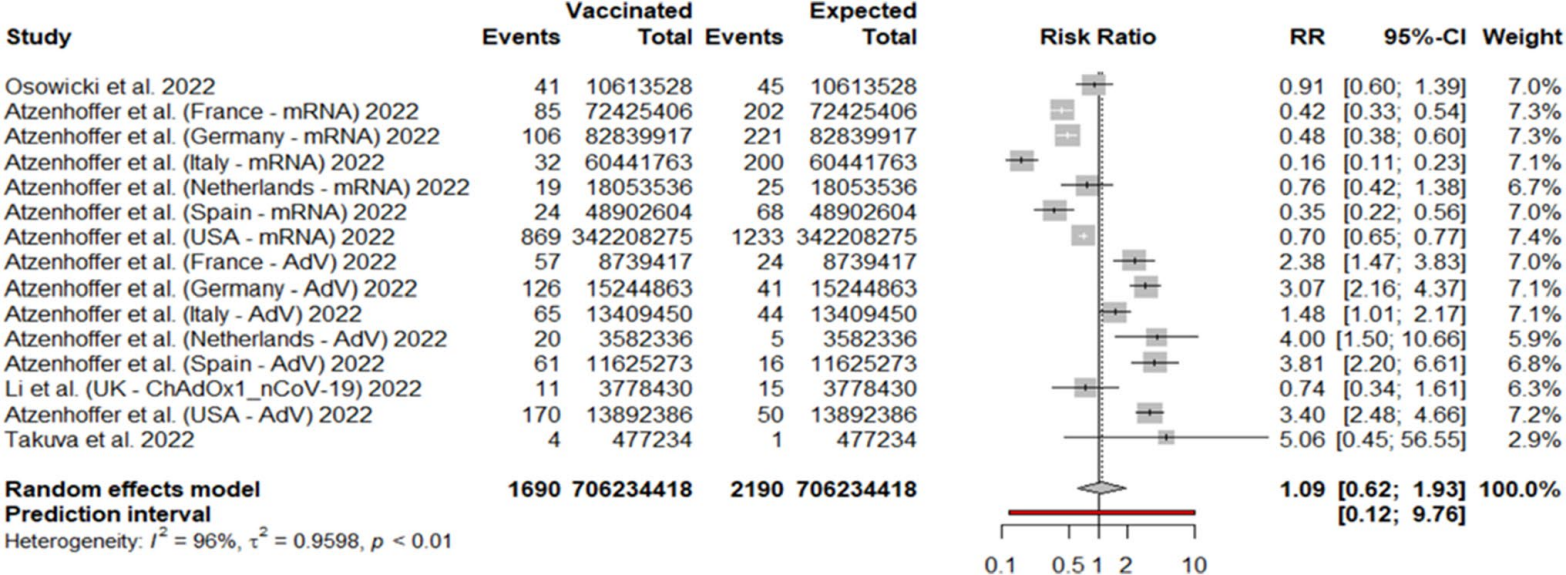
Forest plots of risk ratio between observed and expected GBS linked to COVID-19 vaccine doses administered regardless the vaccine technology; **B** with respect to vector-based vaccines; **C** with respect to mRNA vaccines. For vaccinated, Events: number of GBS cases; Total: number of vaccine doses. For expected, Events: number of expected GBS cases; Total: number of subjects in the population considered. AdV: adenoviral-vectored vaccines

We included 15 cohorts from 5 studies in the analysis of risk between observed and expected GBS cases after COVID-19 vaccination regardless of the vaccine employed. The quantification method for expected cases for each study is summarised in SI. We identified 1690 observed and 2190 expected GBS cases over a total of 706,234,418 vaccine doses. The MH method yielded an RR of 1.09 (95%CI 0.68; 0.90) (Fig. 5).

**Fig. 6:**
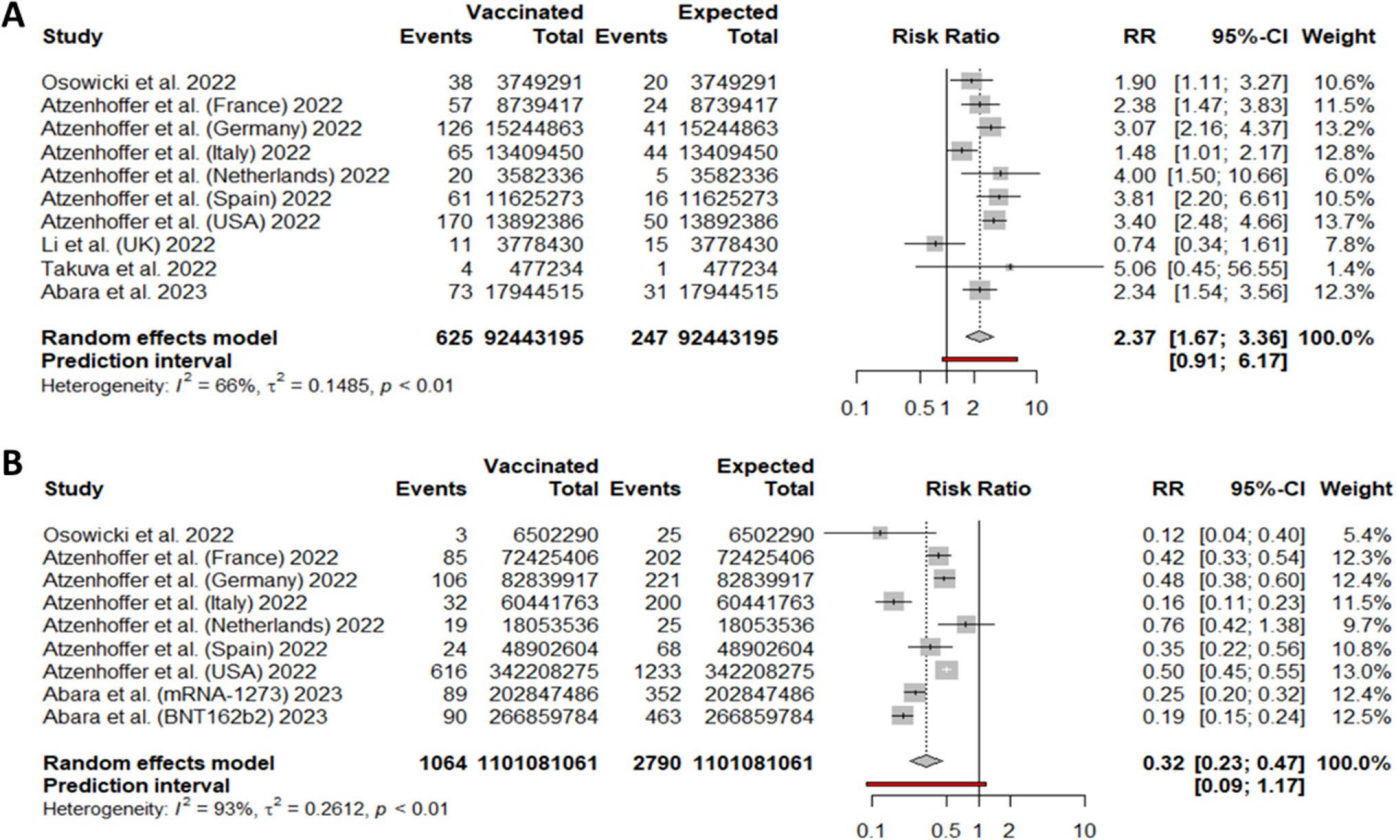
Forest plot about risk ratio between GBS cases linked to COVID-19 vaccine doses administered; **A** with respect to adenoviral-vectored vaccines; **B** with respect to mRNA vaccines. For vaccinated, Events: number of GBS cases; Total: number of vaccine doses. For expected, Events: number of expected GBS cases; Total: number of subjects in the population considered

Regarding GBS after adenovirus-based vaccination, we included ten cohorts from 5 studies. The MH method yielded an RR of 2.37 (95%CI 1.67, 3.36) (Fig. 6A). For GBS after mRNA-based vaccination, we included nine cohorts. The MH method yielded an RR of 0.32 (95%CI 0.23, 0.47) (Fig. 6B).

### GBS mortality per COVID-19 vaccine doses administered and among GBS cases

**Fig. 7:**
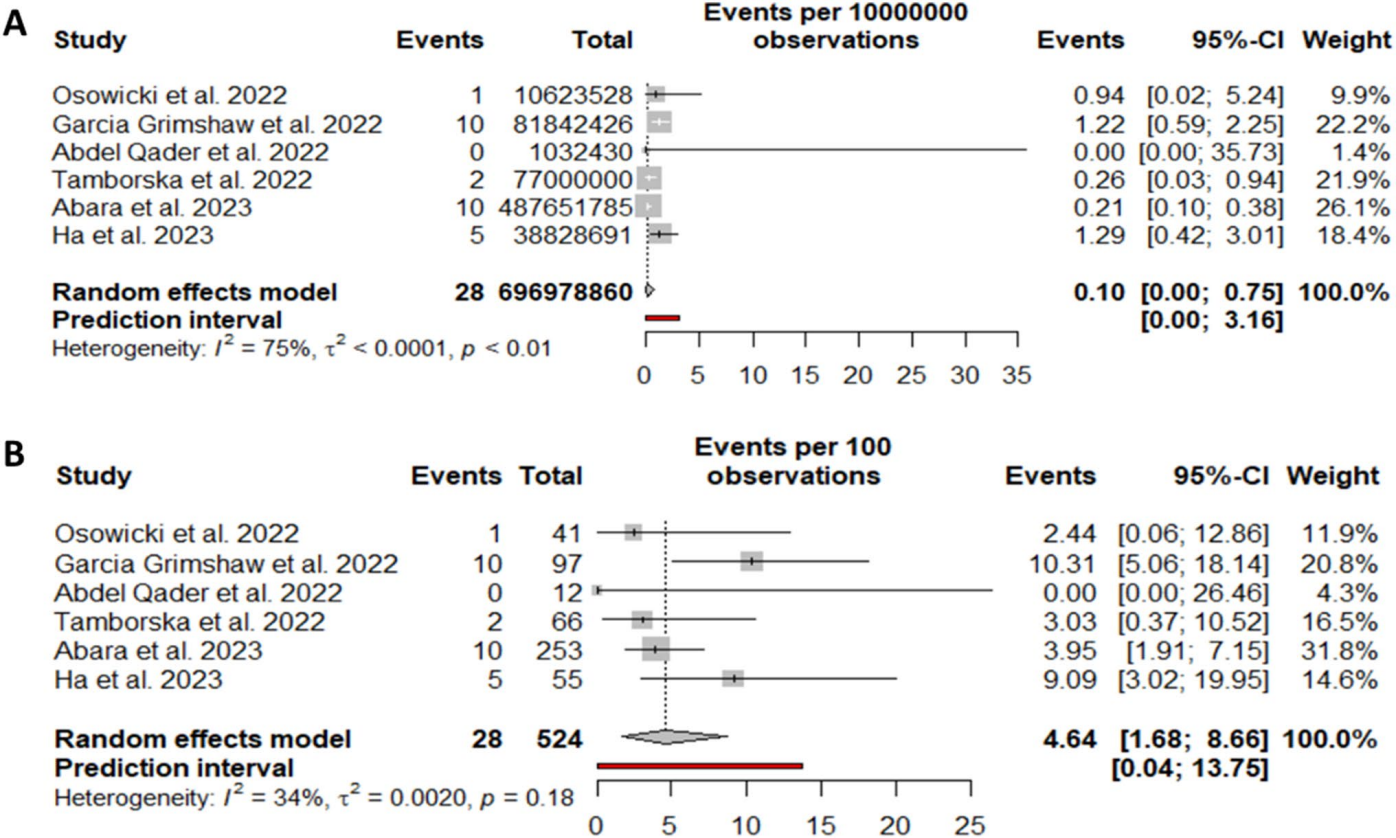
Forest plots of GBS mortality after COVID-19 vaccination. **A** with respect to administered doses; **B** with respect to GBS cases after vaccination. Events: number of deaths; Total: number of total COVID-19 vaccine doses (**A**) and GBS cases (**B**)

We included six cohort studies in the analysis of mortality among people who developed GBS after being vaccinated against COVID-19 regardless of the vaccine technology. We identified 28 deaths in 524 GBS cases for a total of 696,978,860 vaccine doses. The random-effect model proportion yielded 0.10 deaths with GBS per million doses of COVID-19 vaccine (95%CI 0.00; 0.75) (Fig. 7A). Considering the mortality among GBS the random-effect model proportion yielded 4.6 deaths for every 100 GBS cases after COVID-19 vaccination (95%CI 1.23, 5.45) (Fig. 7B).

### Assessment of study quality, publication bias, heterogeneity, and sensitivity analyses

The overall quality of the included studies was deemed high (SI, Table S2). Assessment of funnel plot asymmetry with visual inspection and Egger’s and Peter’s test showed potential asymmetry for the following analyses: all vaccine technologies and adenoviral-vectored incidence, risk ratio between observed and expected for all vaccine technologies. Both FTT and GLMM methods showed similar results, with GLMM giving greater asymmetry in funnel plots and slightly wider prediction intervals (SI, Section 2). Leave-one-out sensitivity analysis was performed for some analyses (SI, Section 2). No single study significantly affected the computed effect size for each outcome, as shown by influence analysis and outlier remotion (SI, Section 2). Baujat plots were also produced when feasible to elucidate the contribution of a single study to the overall random-effect model heterogeneity (SI, Section 2).

## Discussion

This meta-analysis showed, regardless of the vaccine technology, a rate of 1.25 GBS cases per million of COVID-19 vaccine doses. However, the GBS rate for adenovirus-vectored vaccines was five times higher than for mRNA vaccines.

Overall COVID-19 vaccination was not associated with an increased risk of GBS but, disaggregating the data, the GBS risk with adenovirus-vectored vaccines (regardless of whether the vector was simian or human) was 2.4 times increased and about seven times higher compared with mRNA vaccines.

The increased GBS risk with adenovirus-vectored vaccines is not easily explainable. An antecedent adenovirus infection was found to be not more frequent in GBS cases than in controls [40]. Adenoviruses, commonly used to deliver vaccination antigens to the host, are thought to be safe and only a single observational study suggested a link between adenovirus oral vaccination and GBS in a military population [41]. However, 10 out of the 12 GBS-reported cases had also a preceding upper respiratory infection, as a possible GBS trigger, and received multiple vaccines making the association unlikely. Adenovirus-vectored vaccines contain the SARS-CoV-2 gene to codify the spike protein, but the reduced GBS risk associated with mRNA vaccines, which also codify the spike protein, makes unlikely that the spike protein is the causative factor for the increased GBS risk. A non-specific immune activation in susceptible individuals can be hypothesized but, if this is the case, it should occur with all adenovirus-vectored vaccines.

Regarding the reduced GBS risk with the mRNA vaccines, it should be underlined that the SARS-CoV-2 pandemic was characterized by unique and drastic public health measures that, by reducing the circulation of infective agents known to trigger GBS, likely decreased the background risk of GBS. Indeed, most respiratory and common gastrointestinal infections, including *C. jejuni*, decreased significantly in incidence during the pandemic [42, 43]. On the other hand, SARS-CoV-2 infection has been associated in some countries with a higher GBS risk during the early pandemic period and in European cohorts, during the first pandemic wave, the pooled rate of GBS with SARS-CoV-2 infection was 61.3% of the total [44, 45]. A recent study from Israel confirms that GBS risk after Comirnaty vaccine was about 15 times reduced compared with the risk after SARS-CoV-2 infection [46]. These observations suggest that the reduced GBS risk associated with mRNA vaccines represents probably the background GBS risk due to the combined effect of health measures and vaccination.

Our meta-analysis shows that the occurrence of GBS is more than twice as frequent after the first vaccine dose compared to the second one, confirming previous findings [39]. This may be explained by an individual susceptibility triggering GBS with the very first dose and because patients experiencing serious adverse effects from the first dose might have preferred not to undergo the second one. Interestingly, only one patient experiencing GBS after both the first and the second dose of Vaxzevria was reported in the UK [39]. The mortality rate in GBS after COVID-19 vaccination is about 5%, which is in line with the previously reported range (3–10%) [1].

## Conclusions

This meta-analysis confirms an increased, although overall low, risk of GBS following adenovirus-vectored COVID-19 vaccines. The incidence of GBS cases per million vaccinations is lower than the estimated incidence for the 1976/1977 “swine flu” vaccination but is higher than for the modern influenza vaccine. mRNA vaccines were associated with decreased GBS risk, possibly because of a reduction in infections, including SARS-CoV-2, that may be associated with GBS during the vaccination period. Overall, the established epidemiological benefits of COVID-19 vaccination in reducing viral transmission, hospitalization, and deaths, greatly overtake, in our opinion, the risk of developing GBS even after adenovirus-vectored vaccines. How adenovirus-vectored COVID-19 vaccines may trigger GBS is unclear and further studies should investigate the relationship between vaccine technologies and GBS to optimise patients’ safety.

### Supplementary Information

Below is the link to the electronic supplementary material.

Supplementary file1 (DOCX 344 KB)

## Data Availability

Data extracted from included studies, data used for all analyses, and analytic code are available upon reasonable request.

## References

[1] Leonhard SE, Mandarakas MR, Gondim FA (2019). Diagnosis and management of Guillain–Barré syndrome in ten steps. Nat Rev Neurol.

[2] McGrogan A, Madle GC, Seaman HE, De Vries CS (2009). The epidemiology of Guillain–Barré syndrome worldwide. Neuroepidemiology.

[3] Wakerley BR, Uncini A, Yuki N, the GBS Classification Group (2014). Guillain–Barré and Miller Fisher syndromes—new diagnostic classification. Nat Rev Neurol.

[4] Langmuir AD, Bregman DJ, Kurland LT (1984). An epidemiologic and clinical evaluation of Guillain–Barré syndrome reported in association with the administration of Swine influenza vaccines. Am J Epidemiol.

[5] Schonberger LB, Bregman DJ, Sullivan-Bolyai JZ (1979). Guillain–Barre syndrome following vaccination in the national influenza immunization program, United States, 1976–19771. Am J Epidemiol.

[6] Lasky T, Terracciano GJ, Magder L (1998). The Guillain–Barré syndrome and the 1992–1993 and 1993–1994 influenza vaccines. N Engl J Med.

[7] Juurlink DN (2006). Guillain–Barré syndrome after influenza vaccination in adults: a population-based study. Arch Intern Med.

[8] Goud R, Lufkin B, Duffy J (2021). Risk of Guillain–Barré syndrome following recombinant Zoster vaccine in medicare beneficiaries. JAMA Intern Med.

[9] Baxter R, Bakshi N, Fireman B (2013). Lack of association of Guillain–Barre syndrome with vaccinations. Clin Infect Dis.

[10] Lopez Bernal J, Andrews N, Gower C (2021). Effectiveness of Covid-19 vaccines against the B.1.617.2 (Delta) variant. N Engl J Med.

[11] Chagla Z (2021). The BNT162b2 (BioNTech/Pfizer) vaccine had 95% efficacy against COVID-19 ≥7 days after the 2nd dose. Ann Intern Med.

[12] Dagan N, Barda N, Kepten E (2021). BNT162b2 mRNA Covid-19 vaccine in a nationwide mass vaccination setting. N Engl J Med.

[13] EMA (2021) EMA 14 July 2021 COVID-19 vaccine safety update: VAXZEVRIA AstraZeneca AB

[14] Rosenblum HG, Hadler SC, Moulia D, et al (2021) Use of COVID-19 vaccines after reports of adverse events among adult recipients of Janssen (Johnson & Johnson) and mRNA COVID-19 Vaccines (Pfizer-BioNTech and Moderna): update from the advisory committee on immunization Practices—United States, July 2021. MMWR Morb Mortal Wkly Rep 70: 1094–1099. 10.15585/mmwr.mm7032e4 10.15585/mmwr.mm7032e4 PMC8360272 34383735

[15] Patone M, Handunnetthi L, Saatci D (2021). Neurological complications after first dose of COVID-19 vaccines and SARS-CoV-2 infection. Nat Med.

[16] Our World in Data Our World in Data-COVID19 vaccination worldwide

[17] Salmon DA, Proschan M, Forshee R (2013). Association between Guillain–Barré syndrome and influenza A (H1N1) 2009 monovalent inactivated vaccines in the USA: a meta-analysis. The Lancet.

[18] Moher D, Liberati A, Tetzlaff J, Altman DG (2009). Preferred reporting items for systematic reviews and meta-analyses: The PRISMA statement. J Clin Epidemiol.

[19] Wells G, Shea B, O’Connell D, et al The Newcastle-Ottawa Scale (NOS) for assessing the quality if nonrandomized studies in meta-analyses. https://www.ohri.ca/programs/clinical_epidemiology/oxford.asp

[20] Harrer M, Cuijpers P, Furukawa TA, Ebert DD (2021). Doing meta-analysis with R: a hands-on guide.

[21] Chen Y, Chen D, Wang Y, Han Y (2022). Using Freeman–Tukey double arcsine transformation in meta-analysis of single proportions. Aesthetic Plast Surg.

[22] Higgins JPT, Thompson SG (2002). Quantifying heterogeneity in a meta-analysis. Statist Med.

[23] Al Amer FM, Lin L (2021). Empirical assessment of prediction intervals in Cochrane meta-analyses. Eur J Clin Investig.

[24] Peters JL (2006). Comparison of two methods to detect publication bias in meta-analysis. JAMA.

[25] Abara WE, Gee J, Marquez P (2023). Reports of Guillain–Barré syndrome after COVID-19 vaccination in the United States. JAMA Netw Open.

[26] Abdel-Qader DH, Abdel-Qader H, Silverthorne J (2022). Active safety surveillance of four types of COVID-19 vaccines: a national study from Jordan. Clin Drug Investig.

[27] Atzenhoffer M, Auffret M, Pegat A (2022). Guillain–Barré syndrome associated with COVID-19 vaccines: a perspective from spontaneous report data. Clin Drug Investig.

[28] García-Grimshaw M, Galnares-Olalde JA, Bello-Chavolla OY (2022). Incidence of Guillain–Barré syndrome following SARS-CoV -2 immunization: analysis of a nationwide registry of recipients of 81 million doses of seven vaccines. Eur J Neurol.

[29] Gupta A, Paliwal VK, Garg RK (2020). Is COVID-19-related Guillain–Barré syndrome different?. Brain Behav Immun.

[30] Ha J, Park S, Kang H (2023). Real-world data on the incidence and risk of Guillain–Barré syndrome following SARS-CoV-2 vaccination: a prospective surveillance study. Sci Rep.

[31] Koh JS, Hoe RHM, Yong MH (2021). Hospital-based observational study of neurological disorders in patients recently vaccinated with COVID-19 mRNA vaccines. J Neurol Sci.

[32] Li X, Raventós B, Roel E (2022). Association between covid-19 vaccination, SARS-CoV-2 infection, and risk of immune mediated neurological events: population based cohort and self-controlled case series analysis. BMJ.

[33] Osowicki J, Morgan HJ, Harris A (2022). Guillain–Barré syndrome temporally associated with COVID-19 vaccines in Victoria, Australia. Vaccine.

[34] Otero-Losada M, Petrovsky N, Alami A (2023). Disproportionality analysis of adverse neurological and psychiatric reactions with the ChAdOx1 (Oxford-AstraZeneca) and BNT162b2 (Pfizer-BioNTech) COVID-19 vaccines in the United Kingdom. Expert Opin Drug Saf.

[35] Shao S-C, Wang C-H, Chang K-C (2021). Guillain–Barré syndrome associated with COVID-19 vaccination. Emerg Infect Dis.

[36] Shasha D, Bareket R, Sikron FH (2022). Real-world safety data for the Pfizer BNT162b2 SARS-CoV-2 vaccine: historical cohort study. Clin Microbiol Infect.

[37] Takuva S, Takalani A, Seocharan I (2022). Safety evaluation of the single-dose Ad26.COV2.S vaccine among healthcare workers in the Sisonke study in South Africa A phase 3b implementation trial. PLoS Med.

[38] Tamborska AA, Singh B, Leonhard SE (2022). Guillain–Barré syndrome following SARS-CoV-2 vaccination in the UK: a prospective surveillance study. BMJ Neurol Open.

[39] Keh RYS, Scanlon S, Datta-Nemdharry P (2023). COVID-19 vaccination and Guillain–Barré syndrome: analyses using the National Immunoglobulin Database. Brain.

[40] Jacobs BC, Rothbarth PH, Van Der Meché FGA (1998). The spectrum of antecedent infections in Guillain–Barré syndrome: a case–control study. Neurology.

[41] McNeil MM, Paradowska-Stankiewicz I, Miller ER (2019). Adverse events following adenovirus type 4 and type 7 vaccine, live, oral in the vaccine adverse event reporting System (VAERS), United States, October 2011–July 2018. Vaccine.

[42] Huh K, Jung J, Hong J (2021). Impact of nonpharmaceutical interventions on the incidence of respiratory infections during the coronavirus disease 2019 (COVID-19) Outbreak in Korea: A Nationwide Surveillance Study. Clin Infect Dis.

[43] Lee H, Heo N, Kwon D, Ha J (2022). Deciphering changes in the incidence of the Guillain–Barré syndrome during the COVID-19 pandemic: a nationwide time-series correlation study. BMJ Neurol Open.

[44] Censi S, Bisaccia G, Gallina S (2023). Guillain−Barré syndrome and SARS-CoV-2 infection: a systematic review and meta-analysis on a debated issue and evidence for the ‘Italian factor’. Eur J Neurol Ene.

[45] Palaiodimou L, Stefanou M-I, Katsanos AH (2021). Prevalence, clinical characteristics and outcomes of Guillain–Barré syndrome spectrum associated with COVID-19: a systematic review and meta-analysis. Eur J Neurol.

[46] Bishara H, Arbel A, Barnett-Griness O, Bloch S, Cohen S, Najjar-Debbiny R (2023). Association between Guillain–Barré syndrome and COVID-19 infection and vaccination: a population-based nested case-control study. Neurology.

