# Supplementary material for: Guillain–Barré syndrome and COVID-19 vaccination: a systematic review and meta-analysis
Source: J Neurol. 2024 Jan 17;271(3):1063–71. doi: 10.1007/s00415-024-12186-7 (PMC10896967; doi:10.1007/s00415-024-12186-7)

**Guillain-Barré Syndrome and COVID-19 vaccination: a systematic review and meta-analysis**

Stefano Censi^1^, Giandomenico Bisaccia^1^, Sabina Gallina^1^,
Valentina Tomassini^1,2^, Antonino Uncini^1^*

1. Department of Neuroscience, Imaging and Clinical Sciences, “G. d’Annunzio” University of Chieti-Pescara, 66100 Chieti, Italy; [stefano.censi@unich.it](mailto:stefano.censi@unich.it); [giandomenico.bisaccia@unich.it](mailto:giandomenico.bisaccia@unich.it); [uncini@unich.it](mailto:uncini@unich.it), [sabina.gallina@unich.it](mailto:sabina.gallina@unich.it); [valentina.tomassini@unich.it](mailto:valentina.tomassini@unich.it)
2. Clinical Neurology, SS. Annunziata University Hospital, Chieti, Italy

* Corresponding Author

Prof. Antonino Uncini

Department of Neuroscience, Imaging and Clinical Sciences - University “G. d’Annunzio”

Via Luigi Polacchi 11, 66100, Chieti, Italy

+39 0871 3556901

email: [uncini@unich.it](mailto:uncini@unich.it)

**Supplemental Material**

Section 1: studies information (Page 1)

- Table S1 – PRISMA checklist (Page 2)
- Table S2 – Newcastle-Ottawa scale (Page 4)
- Table S3 – Information about included vaccines (Page 5)
- Table S4 – Expected GBS incidence source for Risk Analysis (Page 5)
- Table S5 – GBS Diagnosis criteria for each included cohort study (Page 6)

Section 2: data analysis (Page 7)

**Section 1**

Table S1 – Prisma Checklist

| Section and Topic | Item # | Checklist item | Location where the item is reported? |
| --- | --- | --- | --- |
| TITLE | | |  |
| Title | 1 | Identify the report as a systematic review. | 1 |
| ABSTRACT | | |  |
| Abstract | 2 | See the PRISMA 2020 for Abstracts checklist. | 2 |
| INTRODUCTION | | |  |
| Rationale | 3 | Describe the rationale for the review in the context of existing knowledge. | 3 |
| Objectives | 4 | Provide an explicit statement of the objective(s) or question(s) the review addresses. | 3 |
| METHODS | | |  |
| Eligibility criteria | 5 | Specify the inclusion and exclusion criteria for the review and how studies were grouped for the syntheses. | 4 |
| Information sources | 6 | Specify all databases, registers, websites, organisations, reference lists and other sources searched or consulted to identify studies. Specify the date when each source was last searched or consulted. | 4 |
| Search strategy | 7 | Present the full search strategies for all databases, registers and websites, including any filters and limits used. | 4 |
| Selection process | 8 | Specify the methods used to decide whether a study met the inclusion criteria of the review, including how many reviewers screened each record and each report retrieved, whether they worked independently, and if applicable, details of automation tools used in the process. | 4 |
| Data collection process | 9 | Specify the methods used to collect data from reports, including how many reviewers collected data from each report, whether they worked independently, any processes for obtaining or confirming data from study investigators, and if applicable, details of automation tools used in the process. | 4 |
| Data items | 10a | List and define all outcomes for which data were sought. Specify whether all results that were compatible with each outcome domain in each study were sought (e.g. for all measures, time points, analyses), and if not, the methods used to decide which results to collect. | 4 |
|  | 10b | List and define all other variables for which data were sought (e.g. participant and intervention characteristics, funding sources). Describe any assumptions made about any missing or unclear information. | 4 |
| Study risk of bias assessment | 11 | Specify the methods used to assess risk of bias in the included studies, including details of the tool(s) used, how many reviewers assessed each study and whether they worked independently, and if applicable, details of automation tools used in the process. | 4 |
| Effect measures | 12 | Specify for each outcome the effect measure(s) (e.g. risk ratio, mean difference) used in the synthesis or presentation of results. | 4 |
| Synthesis methods | 13a | Describe the processes used to decide which studies were eligible for each synthesis (e.g. tabulating the study intervention characteristics and comparing against the planned groups for each synthesis (item #5)). | NO |
|  | 13b | Describe any methods required to prepare the data for presentation or synthesis, such as handling of missing summary statistics, or data conversions. | 4 |
|  | 13c | Describe any methods used to tabulate or visually display results of individual studies and syntheses. | 4 |
|  | 13d | Describe any methods used to synthesize results and provide a rationale for the choice(s). If meta-analysis was performed, describe the model(s), method(s) to identify the presence and extent of statistical heterogeneity, and software package(s) used. | 4 |
|  | 13e | Describe any methods used to explore possible causes of heterogeneity among study results (e.g. subgroup analysis, meta-regression). | 4 |
|  | 13f | Describe any sensitivity analyses conducted to assess robustness of the synthesized results. | 4 |
| Reporting bias assessment | 14 | Describe any methods used to assess risk of bias due to missing results in a synthesis (arising from reporting biases). | 4 |
| Certainty assessment | 15 | Describe any methods used to assess certainty (or confidence) in the body of evidence for an outcome. | 4 |
| RESULTS | | |  |
| Study selection | 16a | Describe the results of the search and selection process, from the number of records identified in the search to the number of studies included in the review, ideally using a flow diagram. | 5 |
|  | 16b | Cite studies that might appear to meet the inclusion criteria, but which were excluded, and explain why they were excluded. | NO |
| Study characteristics | 17 | Cite each included study and present its characteristics. | 5 |
| Risk of bias in studies | 18 | Present assessments of risk of bias for each included study. | SupMat |
| Results of individual studies | 19 | For all outcomes, present, for each study: (a) summary statistics for each group (where appropriate) and (b) an effect estimate and its precision (e.g. confidence/credible interval), ideally using structured tables or plots. | 5-8 |
| Results of syntheses | 20a | For each synthesis, briefly summarise the characteristics and risk of bias among contributing studies. | SupMat |
|  | 20b | Present results of all statistical syntheses conducted. If meta-analysis was done, present for each the summary estimate and its precision (e.g. confidence/credible interval) and measures of statistical heterogeneity. If comparing groups, describe the direction of the effect. | 5-8 |
|  | 20c | Present results of all investigations of possible causes of heterogeneity among study results. | 5-8 |
|  | 20d | Present results of all sensitivity analyses conducted to assess the robustness of the synthesized results. | 5-8 |
| Reporting biases | 21 | Present assessments of risk of bias due to missing results (arising from reporting biases) for each synthesis assessed. | 5-8, SupMat |
| Certainty of evidence | 22 | Present assessments of certainty (or confidence) in the body of evidence for each outcome assessed. | 5-8 |
| DISCUSSION | | |  |
| Discussion | 23a | Provide a general interpretation of the results in the context of other evidence. | 8-9 |
|  | 23b | Discuss any limitations of the evidence included in the review. | 8-9 |
|  | 23c | Discuss any limitations of the review processes used. | NO |
|  | 23d | Discuss implications of the results for practice, policy, and future research. | 8-9 |
| OTHER INFORMATION | | |  |
| Registration and protocol | 24a | Provide registration information for the review, including register name and registration number, or state that the review was not registered. | 4 |
|  | 24b | Indicate where the review protocol can be accessed, or state that a protocol was not prepared. | 4 |
|  | 24c | Describe and explain any amendments to information provided at registration or in the protocol. | NO |
| Support | 25 | Describe sources of financial or non-financial support for the review, and the role of the funders or sponsors in the review. | NO |
| Competing interests | 26 | Declare any competing interests of review authors. | 10 |
| Availability of data, code and other materials | 27 | Report which of the following are publicly available and where they can be found: template data collection forms; data extracted from included studies; data used for all analyses; analytic code; any other materials used in the review. | 10 |

*SI: Supplementary Information*

# PRISMA Abstract Checklist

| Topic | No. | Item | Reported? |
| --- | --- | --- | --- |
| TITLE |  |  |  |
| Title | 1 | Identify the report as a systematic review. | Y |
| BACKGROUND |  |  |  |
| Objectives | 2 | Provide an explicit statement of the main objective(s) or question(s) the review addresses. | Y |
| METHODS |  |  |  |
| Eligibility criteria | 3 | Specify the inclusion and exclusion criteria for the review. | N |
| Information sources | 4 | Specify the information sources (e.g. databases, registers) used to identify studies and the date when each was last searched. | N |
| Risk of bias | 5 | Specify the methods used to assess risk of bias in the included studies. | N |
| Synthesis of results | 6 | Specify the methods used to present and synthesize results. | N |
| RESULTS |  |  |  |
| Included studies | 7 | Give the total number of included studies and participants and summarise relevant characteristics of studies. | N |
| Synthesis of results | 8 | Present results for main outcomes, preferably indicating the number of included studies and participants for each. If meta-analysis was done, report the summary estimate and confidence/credible interval. If comparing groups, indicate the direction of the effect (i.e. which group is favoured). | Y |
| DISCUSSION |  |  |  |
| Limitations of evidence | 9 | Provide a brief summary of the limitations of the evidence included in the review (e.g. study risk of bias, inconsistency and imprecision). | Y |
| Interpretation | 10 | Provide a general interpretation of the results and important implications. | Y |
| OTHER |  |  |  |
| Funding | 11 | Specify the primary source of funding for the review. | N |
| Registration | 12 | Provide the register name and registration number. | N |

*From:*  Page MJ, McKenzie JE, Bossuyt PM, Boutron I, Hoffmann TC, Mulrow CD, et al. The PRISMA 2020 statement: an updated guideline for reporting systematic reviews. BMJ 2021;372:n71. doi: 10.1136/bmj.n71

For more information, visit: <http://www.prisma-statement.org/>

Table S2 - NEWCASTLE - OTTAWA QUALITY ASSESSMENT SCALE FOR COHORT STUDIES

| Study (First author et al., year of publication) | Selection | Comparability | Outcome | Overall |
| --- | --- | --- | --- | --- |
| Shasha et al., 2021 | XXXX | XX | XXX | 9/9 |
| Shao et al., 2021 | XXX | - | XXX | 6/7 |
| Koh et al., 2021 | XXX | - | XXX | 6/7 |
| Takuva et al., 2022 | XXX | - | XXX | 6/7 |
| Garcia-Grimshaw et al., 2022 | XXX | - | XXX | 6/7 |
| Abdel-Qader et al., 2022 | XXX | - | XXX | 6/7 |
| Gupta et al., 2022 | XX | - | XXX | 5/7 |
| Osowicki et al., 2022 | XXX | - | XX | 5/7 |
| Atzenhoffer et al., 2022 | XXXX | XX | XXX | 9/9 |
| Li et al., 2022 | XXX | X | XXX | 7/9 |
| Keh et al., 2022 | XXX |  | XXX | 6/7 |
| Otero-Losada et al., 2022 | XXX | - | XXX | 6/7 |
| Tamborska et al., 2022 | XXX | - | XXX | 6/7 |
| Abara et al., 2023 | XXXX | XX | XXX | 9/9 |
| Ha et al., 2023 | XXX | - | XXX | 6/7 |
| Overall score | 47/60 | 7/8 | 44/45 | 98/113 (87%) |

Table S3 – Information about included vaccines

| Manufacturer/WHO EUL holder | Trade name (or name used in papers) | Pre-trade or alternative name | Type/Technology |
| --- | --- | --- | --- |
| Pfizer - BioNTech | Comirnaty | BNT162b2; Tozinameran | mRNA |
| University of Oxford - AstraZenaca | Vaxzevria | AZD1222; ChAdOx1 nCoV-19 | Viral vector |
| Serum Institute of India | Covishield | AZD1222; ChAdOx1 nCoV-19 | Viral vector |
| Janssen | Jcovden | Ad26.COV2.S | Viral vector |
| Moderna Biotech | Spikevax | mRNA-1273; Elosameran | mRNA |
| Sinovac | Coronavac | Sinovac SARS-CoV-2 Vaccine (Vero Cell) | Inactivated |
| Sinopharm/BIBP | Sinopharm BIBP COVID-19 Vaccine | Sinopharm SARS-CoV-2 Vaccine (Vero Cell) | Inactivated |
| Russian Direct Investment Fund | Sputnik V |  | Viral vector |
| Biological E | Corbevax |  | Proteic |

Table S4 - Expected GBS incidence source for Risk Analysis

| First author | Source of expected GBS incidence |
| --- | --- |
| Osowicki et al. | ICD10 coded separations recorded in the state-wide hospital Victorian Admissions Episode Dataset during 2015–2019 (with a 12-month washout period applied), calculated as annual average per 100,000 population in adults. The annual rate was divided by 8.69 to adjust for a 42-day risk window period |
| Frontera et al. | published pre-COVID background incidence rates in the U.S. and incidence rates following acute SARS-CoV-2 infection |
| Atzenhoffer et al. | From published background rates for each country |
| Li et al. | Estimated from the general population cohort |
| Takuva et al. | pre-COVID-19 published GBS background rates |
| Abara | pre-COVID-19 published GBS background rates |
| Woo | Published background rates for the 42-day and 21-day risk windows |

Table S5 – GBS Diagnosis criteria for each included cohort study

| First author | GBS diagnosis criteria |
| --- | --- |
| Shasha et al., 2021 | Not specified – MHMO database |
| Shao et al., 2021 | Brighton criteria |
| Koh et al., 2021 | Brighton criteria |
| Takuva et al., 2022 | Not specified – EVDS database |
| Garcia-Grimshaw et al., 2022 | Asbury criteria |
| Abdel-Qader et al., 2022 | Not specified – PV register |
| Gupta et al., 2022 | Asbury criteria |
| Osowicki et al., 2022 | Not specified – SAEFVIC database |
| Atzenhoffer et al., 2022 | Not specified – VigiBase database |
| Li et al., 2022 | Not specified – AURUM database |
| Keh et al., 2022 | Report by a clinician reviewed by an authorized independent panel |
| Otero-Losada et al., 2022 | Not specified – UK regulatory agency database |
| Tamborska et al., 2022 | Brighton criteria |
| Abara et al., 2023 | Brighton criteria |
| Ha et al., 2023 | Brighton criteria |

Figure S1 – Additional information on studies selection

Shasha et al.
Only reported vaccinated people, not doses. To obtain actual doses:
394.609 total vaccinated people
131.033 had the second shot
233.159 with first shot
233.159-131.033=102.126 are the people with just the first dose
(131.033x2) + 102.126 = 364.192 total doses for included people

Koh et al.
1.398.074 total vaccinated with mRNA vaccines
915.344 had two shots
1.398.074 - 915.344 = 482.730 with only one shot
1.830.688 + 482.730 = 2.313.418 total doses

**Section2: Data analysis**

- R Packages (Page 7)
- Cohorts analysis (Page 8)

**R packages**

Dmetar
Harrer, M., Cuijpers, P., Furukawa, T. & Ebert, D. D. (2019). dmetar: Companion R Package For The Guide 'Doing Meta-Analysis in R'. R package version 0.0.9000. URL <http://dmetar.protectlab.org/>.

Meta
Schwarzer, G., Carpenter, J. R., & Rücker, G. (2015). *Meta-Analysis with R*. Springer International Publishing. https://doi.org/10.1007/978-3-319-21416-0

Tidyverse
Wickham, Hadley, Mara Averick, Jennifer Bryan, Winston Chang, Lucy D’Agostino McGowan, Romain François, Garrett Grolemund, et al. 2019. “Welcome to the tidyverse.” Journal of Open Source Software 4 (43): 1686.

Readxl
Wickham H, Bryan J (2023). readxl: Read Excel Files. https://readxl.tidyverse.org, <https://github.com/tidyverse/readxl>.

Cohort Analysis

**GBS incidence in COVID19 vaccinated people
- Freeman-Tukey Transform (FTT)**

Number of studies: k = 17

Number of observations: o = 1058927070

Number of events: e = 1450

events 95%-CI

Random effects model 1.2451 [0.2129; 2.8341]

Prediction interval [0.0000; 9.6173]

Quantifying heterogeneity:

tau^2 < 0.0001 [0.0001; 0.0001]; tau = 0.0008 [0.0096; 0.0097]

I^2 = 97.9% [97.4%; 98.3%]; H = 6.89 [6.16; 7.72]

Test of heterogeneity:

Q d.f. p-value

760.45 16 < 0.0001

Details on meta-analytical method:

- Inverse variance method

- Restricted maximum-likelihood estimator for tau^2

- Q-Profile method for confidence interval of tau^2 and tau

- Hartung-Knapp adjustment for random effects model (df = 16)

- Prediction interval based on t-distribution (df = 15)

- Freeman-Tukey double arcsine transformation

- Clopper-Pearson confidence interval for individual studies

- Events per 1000000 observations

ASSESSMENT OF PUBLICATION BIAS

Linear regression test of funnel plot asymmetry

Egger et al. method

Test result: t = 2.43, df = 15, p-value = 0.0279

Sample estimates:

bias se.bias intercept se.intercept

5.1136 2.1007 0.0009 0.0001

Details:

- multiplicative residual heterogeneity variance (tau^2 = 36.3412)

- predictor: standard error

- weight: inverse variance

- reference: Egger et al. (1997), BMJ

Peters et al. method

Test result: t = 1.72, df = 15, p-value = 0.1066

Sample estimates:

bias se.bias intercept se.intercept

156.0803 90.9033 0.0014 0.0001

Details:

- multiplicative residual heterogeneity variance (tau^2 < 0.0001)

- predictor: inverse of total sample size

- weight: inverse variance of average event probability

- reference: Peters et al. (2006), JAMA

Identified outliers (random-effects model)

"Mexico - Garcia-Grimshaw et al. 2022", "India - Gupta et al. 2022", "Jordan - Abdel-Qader et al. 2022", "USA - Abara et al. 2023"

Results with outliers removed

Number of studies: k = 13

Number of observations: o = 1058927070

Number of events: e = 1450

events 95%-CI

Random effects model 0.8816 [0.1437; 2.0316]

Prediction interval [0.0000; 6.3530]

Quantifying heterogeneity:

tau^2 < 0.0001 [0.0001; 0.0001]; tau = 0.0006 [0.0091; 0.0096]

I^2 = 93.4% [90.5%; 95.5%]; H = 3.90 [3.24; 4.70]

Test of heterogeneity:

Q d.f. p-value

182.63 12 < 0.0001

INFLUENCE ANALYSIS


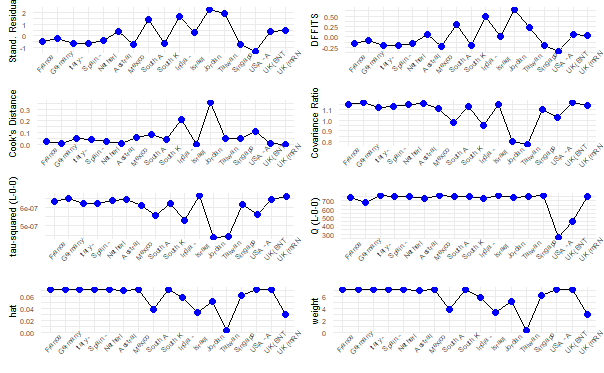


**- General Linear Mixed Model (GLMM)**

Number of studies: k = 17

Number of observations: o = 1058927070

Number of events: e = 1450

events 95%-CI

Random effects model 2.3268 [1.5338; 3.5298]

Prediction interval [0.3998; 13.5403]

Quantifying heterogeneity:

tau^2 = 0.6441; tau = 0.8026; I^2 = 97.9% [97.4%; 98.4%]; H = 6.97 [6.23; 7.80]

Test of heterogeneity:

Q d.f. p-value

Wald 777.22 16 < 0.0001

LRT 800.08 16 < 0.0001

Details on meta-analytical method:

- Random intercept logistic regression model

- Maximum-likelihood estimator for tau^2

- Random effects confidence interval based on t-distribution (df = 16)

- Prediction interval based on t-distribution (df = 15)

- Logit transformation

- Clopper-Pearson confidence interval for individual studies

- Events per 1000000 observations

Identified outliers (random-effects model)

"Garcia-Grimshaw et al. et al. 2022", "Gupta et al. et al. 2022", "Abdel-Qader et al. et al. 2022", "Shao et al. et al. 2021", "Abara et al. et al. 2023"

Results with outliers removed

Number of studies: k = 12

Number of observations: o = 1058927070

Number of events: e = 1450

events 95%-CI

Random effects model 2.0689 [1.5159; 2.8237]

Prediction interval [0.7850; 5.4528]

Quantifying heterogeneity:

tau^2 = 0.1692; tau = 0.4113; I^2 = 94.1% [91.4%; 96.0%]; H = 4.12 [3.42; 4.98]

Test of heterogeneity:

Q d.f. p-value

Wald 186.95 11 < 0.0001

LRT 184.27 11 < 0.0001

ASSESS FOR SMALL STUDIES BIAS

Linear regression test of funnel plot asymmetry

Egger’s method

Test result: t = 0.62, df = 15, p-value = 0.5459

Sample estimates:

bias se.bias intercept se.intercept

1.7655 2.8572 -13.3778 0.3094

Details:

- multiplicative residual heterogeneity variance (tau^2 = 50.5283)

- predictor: standard error

- weight: inverse variance

- reference: Egger et al. (1997), BMJ

Peters’ method

Test result: t = 0.62, df = 15, p-value = 0.5467

Sample estimates:

bias se.bias intercept se.intercept

81778.1692 132598.2871 -13.2314 0.1869

Details:

- multiplicative residual heterogeneity variance (tau^2 = 50.5331)

- predictor: inverse of total sample size

- weight: inverse variance of average event probability

- reference: Peters et al. (2006), JAMA

Funnel plots for FTT and GLMM


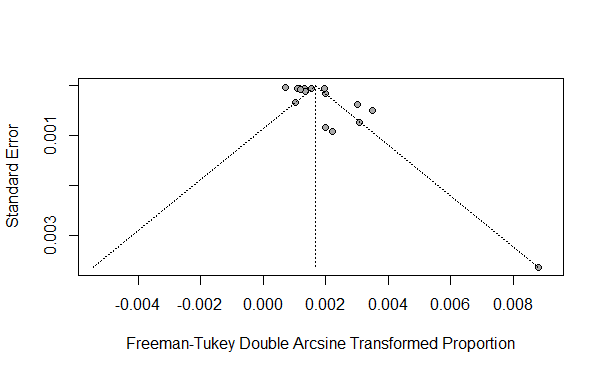

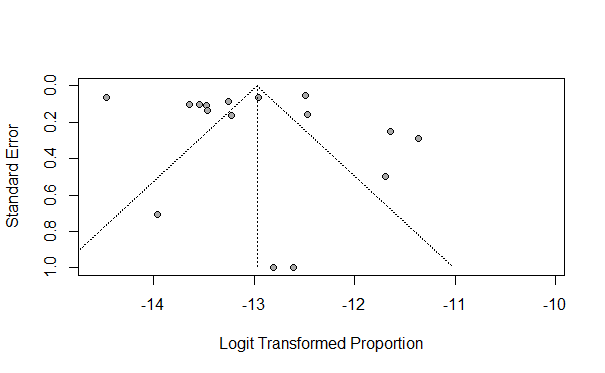


**GBS incidence in COVID19 vaccinated people in Asian countries
FTT**

Number of studies: k = 6

Number of observations: o = 44377532

Number of events: e = 87

events 95%-CI

Random effects model 1.5270 [0.0000; 11.0770]

Quantifying heterogeneity:

tau^2 < 0.0001 [0.0001; 0.0001]; tau = 0.0013 [0.0084; 0.0097]

I^2 = 89.6% [80.1%; 94.6%]; H = 3.10 [2.24; 4.30]

Test of heterogeneity:

Q d.f. p-value

48.10 5 < 0.0001

Details on meta-analytical method:

- Inverse variance method

- Restricted maximum-likelihood estimator for tau^2

- Q-Profile method for confidence interval of tau^2 and tau

- Hartung-Knapp adjustment for random effects model (df = 5)

- Freeman-Tukey double arcsine transformation

- Clopper-Pearson confidence interval for individual studies

- Events per 1000000 observations

Number of studies (k=6) too small to test for small study effects (k.min=10).

**GLMM**Number of studies: k = 6

Number of observations: o = 44377532

Number of events: e = 87

events 95%-CI

Random effects model 3.9063 [1.1495; 13.2744]

Quantifying heterogeneity:

tau^2 = 1.0741; tau = 1.0364; I^2 = 93.9% [89.4%; 96.5%]; H = 4.05 [3.07; 5.36]

Test of heterogeneity:

Q d.f. p-value

Wald 82.20 5 < 0.0001

LRT 58.98 5 < 0.0001

Details on meta-analytical method:

- Random intercept logistic regression model

- Maximum-likelihood estimator for tau^2

- Random effects confidence interval based on t-distribution (df = 5)

- Logit transformation

- Clopper-Pearson confidence interval for individual studies

- Events per 1000000 observations

Number of studies (k=6) too small to test for small study effects (k.min=10).

**GBS incidence in COVID19 vaccinated people in European countries
FTT**

Number of studies: k = 7

Number of observations: o = 433964565

Number of events: e = 968

events 95%-CI

Random effects model 1.8108 [1.0786; 2.7082]

Quantifying heterogeneity:

tau^2 < 0.0001 [0.0001; 0.0001]; tau = 0.0003 [0.0093; 0.0097]

I^2 = 96.0% [93.7%; 97.4%]; H = 4.99 [3.98; 6.25]

Test of heterogeneity:

Q d.f. p-value

149.25 6 < 0.0001

Details on meta-analytical method:

- Inverse variance method

- Restricted maximum-likelihood estimator for tau^2

- Q-Profile method for confidence interval of tau^2 and tau

- Hartung-Knapp adjustment for random effects model (df = 6)

- Freeman-Tukey double arcsine transformation

- Clopper-Pearson confidence interval for individual studies

- Events per 1000000 observations

Number of studies (k=7) too small to test for small study effects (k.min=10).
Identified outliers (random-effects model)

"Otero-Losada et al. (BNT162b2, Chadox1ncov-19) 2023"

Results with outliers removed

Number of studies: k = 6

Number of observations: o = 433964565

Number of events: e = 968

events 95%-CI

Random effects model 1.4972 [1.0520; 2.0104]

Quantifying heterogeneity:

tau^2 < 0.0001 [0.0001; 0.0001]; tau = 0.0002 [0.0108; 0.0096]

I^2 = 84.7% [68.5%; 92.6%]; H = 2.56 [1.78; 3.67]

Test of heterogeneity:

Q d.f. p-value

32.71 5 < 0.0001

**GLMM**Number of studies: k = 7

Number of observations: o = 433964565

Number of events: e = 968

events 95%-CI

Random effects model 1.9571 [1.3624; 2.8114]

Quantifying heterogeneity:

tau^2 = 0.1242; tau = 0.3525; I^2 = 96.1% [93.9%; 97.5%]; H = 5.07 [4.05; 6.33]

Test of heterogeneity:

Q d.f. p-value

Wald 153.97 6 < 0.0001

LRT 153.52 6 < 0.0001

Details on meta-analytical method:

- Random intercept logistic regression model

- Maximum-likelihood estimator for tau^2

- Random effects confidence interval based on t-distribution (df = 6)

- Logit transformation

- Clopper-Pearson confidence interval for individual studies

- Events per 1000000 observations

Number of studies (k=7) too small to test for small study effects (k.min=10).

**GBS Prevalence in COVID19 mRNA vaccinated people**

Number of studies: k = 14

Number of observations: o = 823793743

Number of events: e = 534

events 95%-CI

Random effects model 0.6855 [0.3795; 1.0612]

Prediction interval [0.0001; 2.1824]

Quantifying heterogeneity:

tau^2 < 0.0001 [0.0001; 0.0001]; tau = 0.0003 [0.0091; 0.0096]

I^2 = 92.7% [89.4%; 95.0%]; H = 3.70 [3.08; 4.45]

Test of heterogeneity:

Q d.f. p-value

178.00 13 < 0.0001

Details on meta-analytical method:

- Inverse variance method

- Restricted maximum-likelihood estimator for tau^2

- Q-Profile method for confidence interval of tau^2 and tau

- Hartung-Knapp adjustment for random effects model (df = 13)

- Prediction interval based on t-distribution (df = 12)

- Freeman-Tukey double arcsine transformation

- Clopper-Pearson confidence interval for individual studies

- Events per 1000000 observations

Identified outliers (random-effects model)

"Abara et al. (USA, mRNA-1273) 2023", "Abara et al. (USA, BNT162b2) 2023", "Garcia-Grimshaw et al. (Mexico) 2022"

Results with outliers removed

Number of studies: k = 11

Number of observations: o = 823793743

Number of events: e = 534

events 95%-CI

Random effects model 0.7443 [0.4276; 1.1294]

Prediction interval [0.0570; 1.9640]

Quantifying heterogeneity:

tau^2 < 0.0001 [0.0001; 0.0001]; tau = 0.0002 [0.0108; 0.0095]

I^2 = 81.3% [67.6%; 89.2%]; H = 2.31 [1.76; 3.04]

Test of heterogeneity:

Q d.f. p-value

53.41 10 < 0.0001

ASSESS FOR SMALL STUDIES BIAS

Linear regression test of funnel plot asymmetry

Egger’s method

Test result: t = 2.49, df = 12, p-value = 0.0287

Sample estimates:

bias se.bias intercept se.intercept

3.4386 1.3836 0.0006 0.0001

Details:

- multiplicative residual heterogeneity variance (tau^2 = 9.7927)

- predictor: standard error

- weight: inverse variance

- reference: Egger et al. (1997), BMJ

Peters’ method

Test result: t = 1.22, df = 12, p-value = 0.2451

Sample estimates:

bias se.bias intercept se.intercept

490.4676 401.2703 0.0009 0.0001

Details:

- multiplicative residual heterogeneity variance (tau^2 < 0.0001)

- predictor: inverse of total sample size

- weight: inverse variance of average event probability

- reference: Peters et al. (2006), JAMA

INFLUENCE ANALYSIS


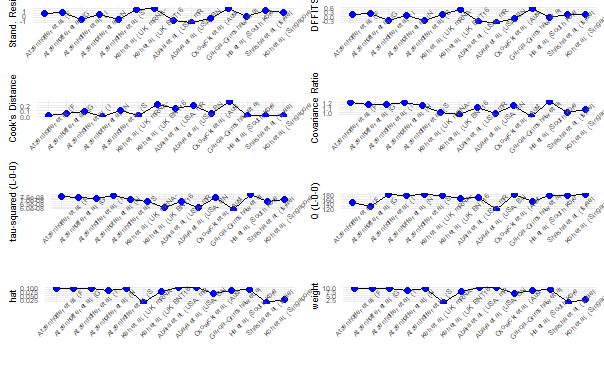


**GLMM**
Number of studies: k = 14

Number of observations: o = 823793743

Number of events: e = 534

events 95%-CI

Random effects model 0.8327 [0.5864; 1.1824]

Prediction interval [0.2420; 2.8655]

Quantifying heterogeneity:

tau^2 = 0.2954; tau = 0.5435; I^2 = 93.2% [90.2%; 95.3%]; H = 3.83 [3.20; 4.59]

Test of heterogeneity:

Q d.f. p-value

Wald 190.72 13 < 0.0001

LRT 185.14 13 < 0.0001

Details on meta-analytical method:

- Random intercept logistic regression model

- Maximum-likelihood estimator for tau^2

- Random effects confidence interval based on t-distribution (df = 13)

- Prediction interval based on t-distribution (df = 12)

- Logit transformation

- Clopper-Pearson confidence interval for individual studies

- Events per 1000000 observations

Identified outliers (random-effects model)

"Keh et al. (UK, BNT162b2) et al. 2022", "Abara et al. (USA, mRNA-1273) et al. 2023", "Abara et al. (USA, BNT162b2) et al. 2023", "Garcia-Grimshaw et al. (Mexico) et al. 2022"

Results with outli

Number of studies: k = 10

Number of observations: o = 823793743

Number of events: e = 534

events 95%-CI

Random effects model 0.8444 [0.6035; 1.1814]

Prediction interval [0.3576; 1.9939]

Quantifying heterogeneity:

tau^2 = 0.1168; tau = 0.3418; I^2 = 77.1% [58.0%; 87.6%]; H = 2.09 [1.54; 2.8]

Test of heterogeneity:

Q d.f. p-value

Wald 39.37 9 < 0.0001

LRT 43.55 9 < 0.0001

ASSESS SMALL STUDIES BIAS
Linear regression test of funnel plot asymmetry

Test result: t = 0.65, df = 12, p-value = 0.5276

Sample estimates:

bias se.bias intercept se.intercept

1.3217 2.0317 -14.2591 0.3290

Details:

- multiplicative residual heterogeneity variance (tau^2 = 15.3518)

- predictor: standard error

- weight: inverse variance

- reference: Egger et al. (1997), BMJ

Linear regression test of funnel plot asymmetry

Test result: t = 0.73, df = 12, p-value = 0.4774

Sample estimates:

bias se.bias intercept se.intercept

666066.4122 908138.3661 -14.0976 0.1714

Details:

- multiplicative residual heterogeneity variance (tau^2 = 15.2114)

- predictor: inverse of total sample size

- weight: inverse variance of average event probability

- reference: Peters et al. (2006), JAMA

Funnel plots for both FTT and GLMM


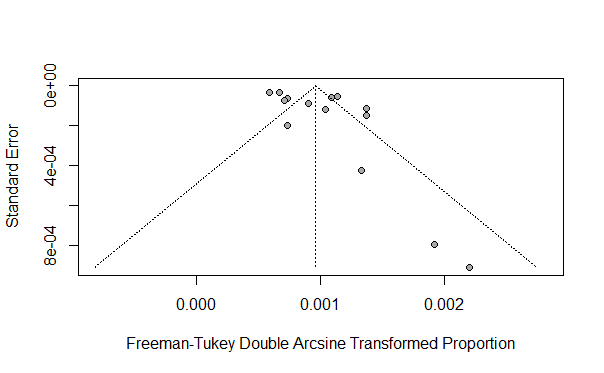

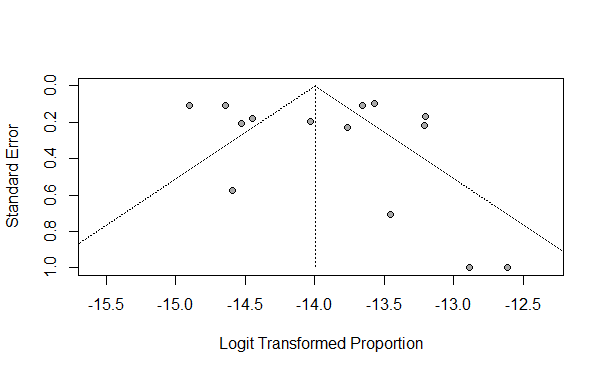


**GBS Prevalence in Comirnaty vaccinated people**

**FTT**

Number of studies: k = 5

Number of observations: o = 648450937

Number of events: e = 419

events 95%-CI

Random effects model 0.6207 [0.1189; 1.3943]

Quantifying heterogeneity:

tau^2 < 0.0001 [0.0001; 0.0001]; tau = 0.0003 [0.0092; 0.0097]

I^2 = 95.6% [92.3%; 97.5%]; H = 4.79 [3.61; 6.35]

Test of heterogeneity:

Q d.f. p-value

91.76 4 < 0.0001

Details on meta-analytical method:

- Inverse variance method

- Restricted maximum-likelihood estimator for tau^2

- Q-Profile method for confidence interval of tau^2 and tau

- Freeman-Tukey double arcsine transformation

- Clopper-Pearson confidence interval for individual studies

- Events per 1000000 observations

**GLMM**

Number of studies: k = 5

Number of observations: o = 648450937

Number of events: e = 419

events 95%-CI

Random effects model 0.7652 [0.4634; 1.2637]

Quantifying heterogeneity:

tau^2 = 0.4212; tau = 0.6490; I^2 = 95.5% [92.1%; 97.5%]; H = 4.74 [3.57; 6.29]

Test of heterogeneity:

Q d.f. p-value

Wald 89.81 4 < 0.0001

LRT 91.99 4 < 0.0001

Details on meta-analytical method:

- Random intercept logistic regression model

- Maximum-likelihood estimator for tau^2

- Logit transformation

- Clopper-Pearson confidence interval for individual studies

- Events per 1000000 observations

**SPIKEVAX**

FTT

Number of studies: k = 3

Number of observations: o = 253796319

Number of events: e = 136

events 95%-CI

Random effects model 0.6634 [0.2711; 1.2065]

Quantifying heterogeneity:

tau^2 < 0.0001 [0.0001; 0.0001]; tau = 0.0002 [0.0101; 0.0097]

I^2 = 87.3% [64.0%; 95.5%]; H = 2.81 [1.67; 4.73]

Test of heterogeneity:

Q d.f. p-value

15.75 2 0.0004

Details on meta-analytical method:

- Inverse variance method

- Restricted maximum-likelihood estimator for tau^2

- Q-Profile method for confidence interval of tau^2 and tau

- Freeman-Tukey double arcsine transformation

- Clopper-Pearson confidence interval for individual studies

- Events per 1000000 observations

**GLMM**

Number of studies: k = 3

Number of observations: o = 253796319

Number of events: e = 136

events 95%-CI

Random effects model 0.6651 [0.4148; 1.0663]

Prediction interval [0.0032; 139.0124]

Quantifying heterogeneity:

tau^2 = 0.1188; tau = 0.3447; I^2 = 88.6% [68.4%; 95.9%]; H = 2.96 [1.78; 4.92]

Test of heterogeneity:

Q d.f. p-value

Wald 17.50 2 0.0002

LRT 15.80 2 0.0004

Details on meta-analytical method:

- Random intercept logistic regression model

- Maximum-likelihood estimator for tau^2

- Prediction interval based on t-distribution (df = 1)

- Logit transformation

- Clopper-Pearson confidence interval for individual studies

- Events per 1000000 observations

**ADENOVECTOR-BASED**

Number of studies: k = 11

Number of observations: o = 127355745

Number of events: e = 806

events 95%-CI

Random effects model 3.9299 [2.5447; 5.5366]

Prediction interval [0.3926; 9.8430]

Quantifying heterogeneity:

tau^2 < 0.0001 [0.0001; 0.0001]; tau = 0.0004 [0.0108; 0.0095]

I^2 = 81.0% [67.0%; 89.1%]; H = 2.29 [1.74; 3.02]

Test of heterogeneity:

Q d.f. p-value

52.64 10 < 0.0001

Details on meta-analytical method:

- Inverse variance method

- Restricted maximum-likelihood estimator for tau^2

- Q-Profile method for confidence interval of tau^2 and tau

- Hartung-Knapp adjustment for random effects model (df = 10)

- Prediction interval based on t-distribution (df = 9)

- Freeman-Tukey double arcsine transformation

- Clopper-Pearson confidence interval for individual studies

- Events per 1000000 observations

No outliers detected (random-effects model).

ASSESSMENT FOR PUBLICATION BIAS

Linear regression test of funnel plot asymmetry

Egger’s method

Test result: t = 0.27, df = 9, p-value = 0.7916

Sample estimates:

bias se.bias intercept se.intercept

0.3862 1.4189 0.0025 0.0002

Details:

- multiplicative residual heterogeneity variance (tau^2 = 5.8009)

- predictor: standard error

- weight: inverse variance

- reference: Egger et al. (1997), BMJ

Peters’ method

Test result: t = 2.26, df = 9, p-value = 0.0502

Sample estimates:

bias se.bias intercept se.intercept

117.1134 51.8384 0.0026 0.0001

Details:

- multiplicative residual heterogeneity variance (tau^2 < 0.0001)

- predictor: inverse of total sample size

- weight: inverse variance of average event probability

- reference: Peters et al. (2006), JAMA

**
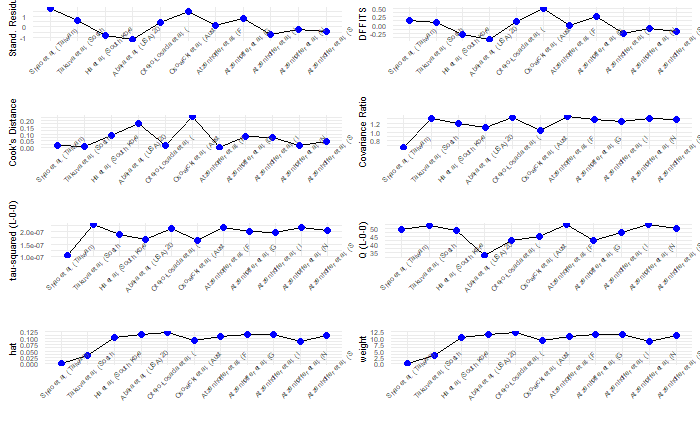
**

**GLMM**Number of studies: k = 11

Number of observations: o = 127355745

Number of events: e = 806

events 95%-CI

Random effects model 6.0990 [4.9643; 7.4931]

Prediction interval [3.3678; 11.0450]

Quantifying heterogeneity:

tau^2 = 0.0604; tau = 0.2457; I^2 = 80.7% [66.5%; 88.9%]; H = 2.28 [1.73;3.01]

Test of heterogeneity:

Q d.f. p-value

Wald 51.91 10 < 0.0001

LRT 51.83 10 < 0.0001

Details on meta-analytical method:

- Random intercept logistic regression model

- Maximum-likelihood estimator for tau^2

- Random effects confidence interval based on t-distribution (df = 10)

- Prediction interval based on t-distribution (df = 9)

- Logit transformation

- Clopper-Pearson confidence interval for individual studies

- Events per 1000000 observations

Identified outliers (random-effects model)

"Shao et al. (Taiwan) et al.2021"

Results with outliers removed

Number of studies: k = 10

Number of observations: o = 127355745

Number of events: e = 806

events 95%-CI

Random effects model 6.0534 [4.9138; 7.4573]

Prediction interval [3.3116; 11.0650]

Quantifying heterogeneity:

tau^2 = 0.0599; tau = 0.2448; I^2 = 81.0% [66.1%; 89.4%]; H = 2.29 [1.72; 3.07]

Test of heterogeneity:

Q d.f. p-value

Wald 47.39 9 < 0.0001

LRT 49.28 9 < 0.0001

ASSESS SMALL STUDIES BIAS

Linear regression test of funnel plot asymmetry

Egger’s method

Test result: t = -0.29, df = 9, p-value = 0.7795

Sample estimates:

bias se.bias intercept se.intercept

-0.4000 1.3864 -11.8983 0.1620

Details:

- multiplicative residual heterogeneity variance (tau^2 = 5.7150)

- predictor: standard error

- weight: inverse variance

- reference: Egger et al. (1997), BMJ

Peters’ method

Test result: t = 0.94, df = 9, p-value = 0.3703

Sample estimates:

bias se.bias intercept se.intercept

39420.2167 41806.9794 -11.9441 0.0809

Details:

- multiplicative residual heterogeneity variance (tau^2 = 5.2493)

- predictor: inverse of total sample size

- weight: inverse variance of average event probability

- reference: Peters et al. (2006), JAMA

FUNNEL PLOTS (FTT and GLMM)


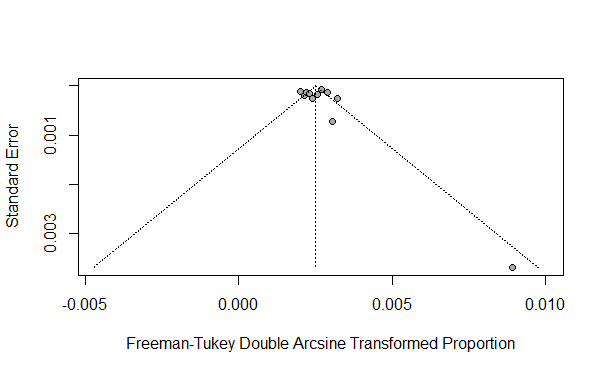

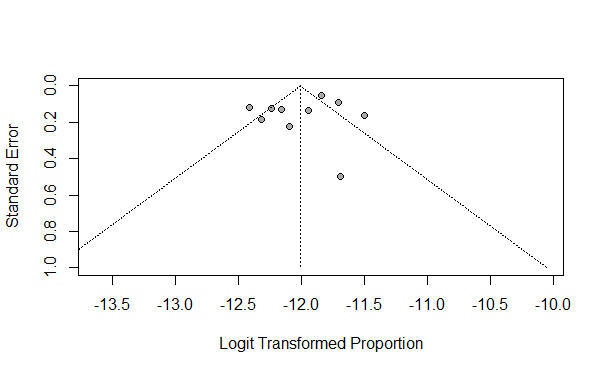


**VAXZEVRIA**

Number of studies: k = 8

Number of observations: o = 227858491

Number of events: e = 1440

events 95%-CI

Random effects model 2.2275 [0.0000; 7.8788]

Prediction interval [0.0000; 24.2104]

Quantifying heterogeneity:

tau^2 < 0.0001 [0.0001; 0.0001]; tau = 0.0011 [0.0097; 0.0098]

I^2 = 99.3% [99.1%; 99.4%]; H = 11.74 [10.42; 13.24]

Test of heterogeneity:

Q d.f. p-value

965.51 7 < 0.0001

Details on meta-analytical method:

- Inverse variance method

- Restricted maximum-likelihood estimator for tau^2

- Q-Profile method for confidence interval of tau^2 and tau

- Hartung-Knapp adjustment for random effects model (df = 7)

- Prediction interval based on t-distribution (df = 6)

- Freeman-Tukey double arcsine transformation

- Clopper-Pearson confidence interval for individual studies

- Events per 1000000 observations

Identified outliers (random-effects model)

"Garcia Grimshaw et al. 2022", "Atzenhoffer et al. 2022"

Results with outliers removed

Number of studies: k = 6

Number of observations: o = 227858491

Number of events: e = 1440


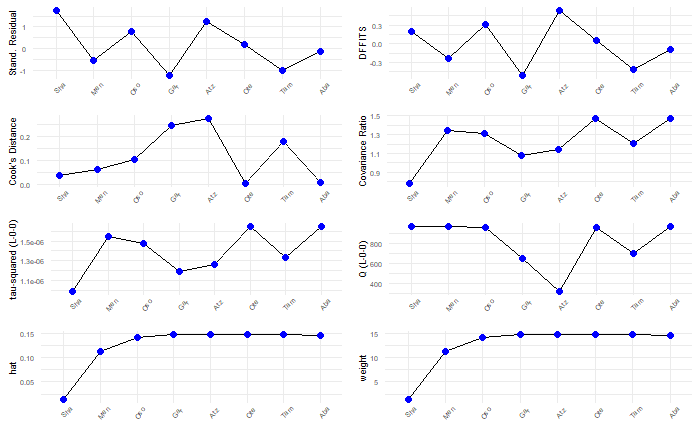
 events 95%-CI

Random effects model 2.2037 [0.0000; 9.5253]

Prediction interval [0.0000; 29.4577]

Quantifying heterogeneity:

tau^2 < 0.0001 [0.0001; 0.0001]; tau = 0.0011 [0.0094; 0.0097]

I^2 = 97.5% [96.1%; 98.4%]; H = 6.30 [5.09; 7.80]

Test of heterogeneity:

Q d.f. p-value

198.43 5 < 0.0001

**
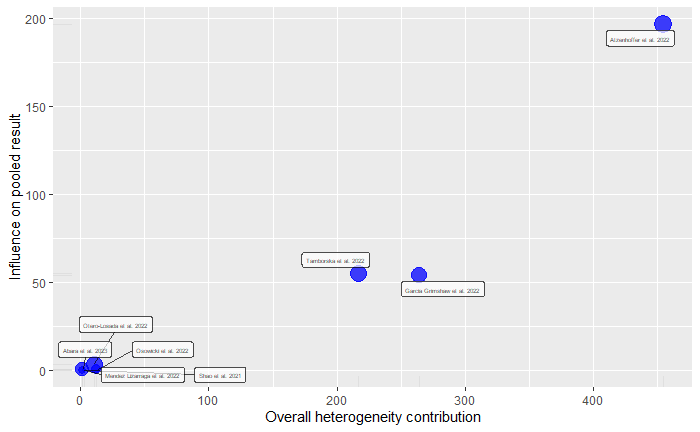
**

**GLMM**Number of studies: k = 8

Number of observations: o = 227858491

Number of events: e = 1440

events 95%-CI

Random effects model 4.2351 [1.7904; 10.0179]

Prediction interval [0.3381; 53.0415]

Quantifying heterogeneity:

tau^2 = 0.9346; tau = 0.9667; I^2 = 98.8% [98.5%; 99.1%]; H = 9.27 [8.05; 10.66]

Test of heterogeneity:

Q d.f. p-value

Wald 600.96 7 < 0.0001

LRT 900.97 7 < 0.0001

Details on meta-analytical method:

- Random intercept logistic regression model

- Maximum-likelihood estimator for tau^2

- Random effects confidence interval based on t-distribution (df = 7)

- Prediction interval based on t-distribution (df = 6)

- Logit transformation

- Clopper-Pearson confidence interval for individual studies

- Events per 1000000 observations

Identified outliers (random-effects model)

"Garcia Grimshaw et al. 2022", "Atzenhoffer et al. 2022"

Results with outliers removed

Number of studies: k = 6

Number of observations: o = 227858491

Number of events: e = 1440

events 95%-CI

Random effects model 4.4615 [1.8353; 10.8455]

Prediction interval [0.4809; 41.3894]

Quantifying heterogeneity:

tau^2 = 0.5243; tau = 0.7241; I^2 = 96.6% [94.6%; 97.9%]; H = 5.42 [4.29; 6.85]

Test of heterogeneity:

Q d.f. p-value

Wald 146.86 5 < 0.0001

LRT 187.21 5 < 0.0001


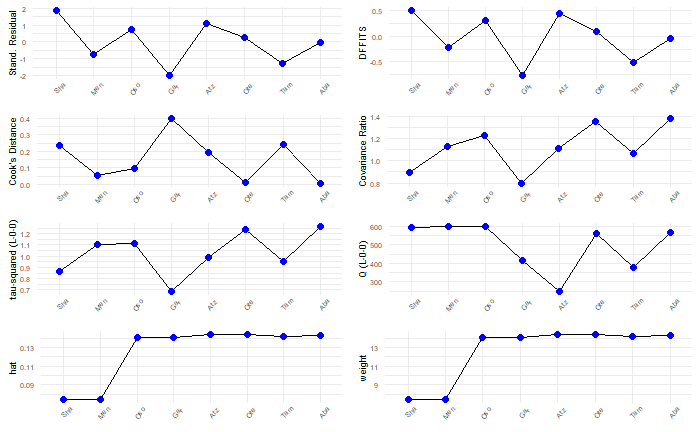


**JCOVDEN**

Number of studies: k = 4

Number of observations: o = 27979000

Number of events: e = 267

events 95%-CI

Random effects model 7.9174 [4.5939; 12.0880]

Quantifying heterogeneity:

tau^2 < 0.0001 [0.0001; 0.0001]; tau = 0.0006 [0.0106; 0.0097]

I^2 = 86.7% [68.0%; 94.5%]; H = 2.75 [1.77; 4.27]

Test of heterogeneity:

Q d.f. p-value

22.61 3 < 0.0001

Details on meta-analytical method:

- Inverse variance method

- Restricted maximum-likelihood estimator for tau^2

- Q-Profile method for confidence interval of tau^2 and tau

- Freeman-Tukey double arcsine transformation

- Clopper-Pearson confidence interval for individual studies

- Events per 1000000 observations

No outliers detected (random-effects model).
Number of studies (k=4) too small to test for small study effects (k.min=10)

**GLMM**

Number of studies: k = 4

Number of observations: o = 27979000

Number of events: e = 267

events 95%-CI

Random effects model 8.1434 [4.2508; 15.6004]

Prediction interval [1.7062; 38.8660]

Quantifying heterogeneity:

tau^2 = 0.0902; tau = 0.3004; I^2 = 85.9% [65.5%; 94.2%]; H = 2.66 [1.70; 4.17]

Test of heterogeneity:

Q d.f. p-value

Wald 21.30 3 < 0.0001

LRT 23.09 3 < 0.0001

Details on meta-analytical method:

- Random intercept logistic regression model

- Maximum-likelihood estimator for tau^2

- Random effects confidence interval based on t-distribution (df = 3)

- Prediction interval based on t-distribution (df = 2)

- Logit transformation

- Clopper-Pearson confidence interval for individual studies

- Events per 1000000 observations

No outliers detected (random-effects model).
Number of studies (k=4) too small to test for small study effects (k.min=10).

**MORTALITY WITH RESPECT TO ADMINISTERED DOSES**

FTT

Number of studies: k = 6

Number of observations: o = 696978860

Number of events: e = 28

events 95%-CI

Random effects model 0.0961 [0.0000; 0.7491]

Prediction interval [0.0000; 3.1646]

Quantifying heterogeneity:

tau^2 < 0.0001 [0.0000; 0.0001]; tau = 0.0001 [0.0000; 0.0096]

I^2 = 75.1% [43.6%; 89.0%]; H = 2.00 [1.33; 3.01]

Test of heterogeneity:

Q d.f. p-value

20.06 5 0.0012

Details on meta-analytical method:

- Inverse variance method

- Restricted maximum-likelihood estimator for tau^2

- Q-Profile method for confidence interval of tau^2 and tau

- Hartung-Knapp adjustment for random effects model (df = 5)

- Prediction interval based on t-distribution (df = 4)

- Freeman-Tukey double arcsine transformation

- Clopper-Pearson confidence interval for individual studies

- Events per 10000000 observations

No outliers detected (random-effects model).
Number of studies (k=6) too small to test for small study effects (k.min=10).


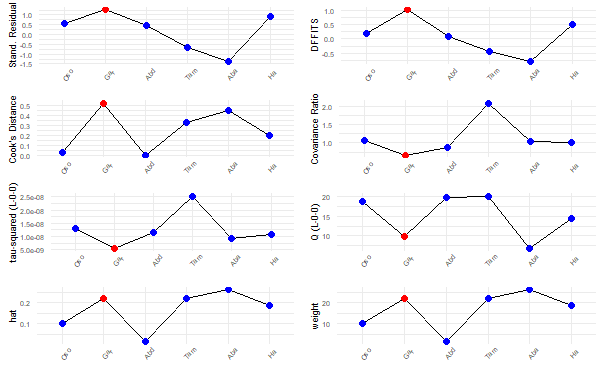


**GLMM**Number of studies: k = 6

Number of observations: o = 696978860

Number of events: e = 28

events 95%-CI

Random effects model 0.5316 [0.1989; 1.4204]

Prediction interval [0.0555; 5.0948]

Quantifying heterogeneity:

tau^2 = 0.5165; tau = 0.7187; I^2 = 76.3% [46.8%; 89.4%]; H = 2.05 [1.37; 3.07]

Test of heterogeneity:

Q d.f. p-value

Wald 21.07 5 0.0008

LRT 20.40 5 0.0010

Details on meta-analytical method:

- Random intercept logistic regression model

- Maximum-likelihood estimator for tau^2

- Random effects confidence interval based on t-distribution (df = 5)

- Prediction interval based on t-distribution (df = 4)

- Logit transformation

- Clopper-Pearson confidence interval for individual studies

- Continuity correction of 0.5 in studies with zero cell frequencies

(only used to calculate individual study results)

- Events per 10000000 observations

No outliers detected (random-effects model).
Number of studies (k=6) too small to test for small study effects (k.min=10)

**MORTALITY WITH RESPECT TO GBS CASES**

Number of studies: k = 6

Number of observations: o = 524

Number of events: e = 28

events 95%-CI

Random effects model 4.6392 [1.6754; 8.6639]

Prediction interval [0.0405; 13.7462]

Quantifying heterogeneity:

tau^2 = 0.0020 [0.0000; 0.0296]; tau = 0.0448 [0.0000; 0.1720]

I^2 = 33.6% [0.0%; 73.3%]; H = 1.23 [1.00; 1.94]

Test of heterogeneity:

Q d.f. p-value

7.53 5 0.1840

Details on meta-analytical method:

- Inverse variance method

- Restricted maximum-likelihood estimator for tau^2

- Q-Profile method for confidence interval of tau^2 and tau

- Hartung-Knapp adjustment for random effects model (df = 5)

- Prediction interval based on t-distribution (df = 4)

- Freeman-Tukey double arcsine transformation

- Clopper-Pearson confidence interval for individual studies

- Events per 100 observations

No outliers detected (random-effects model).

**GLMM**

Number of studies: k = 6

Number of observations: o = 524

Number of events: e = 28

events 95%-CI

Random effects model 5.2632 [2.7220; 9.9347]

Prediction interval [1.6234; 15.7573]

Quantifying heterogeneity:

tau^2 = 0.1200; tau = 0.3464; I^2 = 35.0% [0.0%; 74.0%]; H = 1.24 [1.00; 1.96]

Test of heterogeneity:

Q d.f. p-value

Wald 7.69 5 0.1741

LRT 9.09 5 0.1055

Details on meta-analytical method:

- Random intercept logistic regression model

- Maximum-likelihood estimator for tau^2

- Random effects confidence interval based on t-distribution (df = 5)

- Prediction interval based on t-distribution (df = 4)

- Logit transformation

- Clopper-Pearson confidence interval for individual studies

- Continuity correction of 0.5 in studies with zero cell frequencies

(only used to calculate individual study results)

- Events per 100 observations

No outliers detected (random-effects model).

**GBS risk with first vs second dose**

Number of studies: k = 5

Number of observations: o = 106180721

Number of events: e = 125

RR 95%-CI z p-value

Random effects model 2.6011 [0.4249; 15.9211] 1.03 0.3011

Quantifying heterogeneity:

tau^2 = 2.9202 [0.3624; 36.3955]; tau = 1.7089 [0.6020; 6.0329]

I^2 = 78.7% [49.2%; 91.1%]; H = 2.17 [1.40; 3.35]

Test of heterogeneity:

Q d.f. p-value

18.80 4 0.0009

Details on meta-analytical method:

- Inverse variance method

- Paule-Mandel estimator for tau^2

- Q-Profile method for confidence interval of tau^2 and tau

- Continuity correction of 0.5 in studies with zero cell frequencies

No outliers detected (random-effects model).


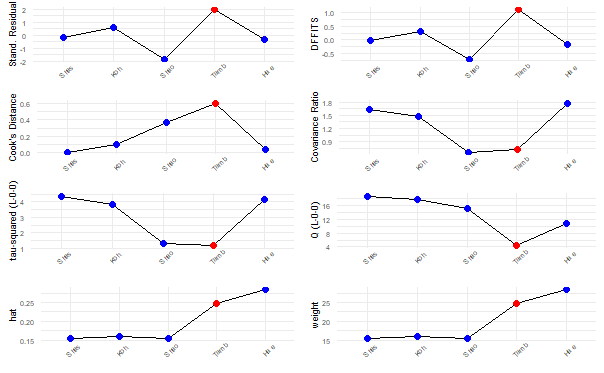


**GBS risk between observed and expected GBS cases for all COVID-19 vaccines**

Number of studies: k = 15

Number of observations: o = 1412468836

Number of events: e = 3879.69

RR 95%-CI t p-value

Random effects model 1.0894 [0.6156; 1.9279] 0.32 0.7525

Prediction interval [0.1215; 9.7647]

Quantifying heterogeneity:

tau^2 = 0.9598 [0.4743; 2.5745]; tau = 0.9797 [0.6887; 1.6045]

I^2 = 95.9% [94.5%; 97.0%]; H = 4.96 [4.27; 5.75]

Test of heterogeneity:

Q d.f. p-value

344.06 14 < 0.0001

Details on meta-analytical method:

- Inverse variance method

- Paule-Mandel estimator for tau^2

- Q-Profile method for confidence interval of tau^2 and tau

- Hartung-Knapp adjustment for random effects model (df = 14)

- Prediction interval based on t-distribution (df = 13)

Identified outliers (random-effects model)

"Atzenhoffer et al. (France - mRNA) 2022", "Atzenhoffer et al. (Germany - mRNA), "Atzenhoffer et al. (Italy - mRNA), "Atzenhoffer et al. (Spain - mRNA), "Atzenhoffer et al. (Germany - AdV)", "Atzenhoffer et al. (Spain - AdV)", "Atzenhoffer et al. (USA - AdV) 2022"

Results with outliers removed

Number of studies: k = 8

Number of observations: o = 1412468836

Number of events: e = 3879.69

RR 95%-CI t p-value

Random effects model 1.2521 [0.7153; 2.1917] 0.95 0.3740

Prediction interval [0.2763; 5.6748]

Quantifying heterogeneity:

tau^2 = 0.3254 [0.0755; 2.1419]; tau = 0.5704 [0.2747; 1.4635]

I^2 = 86.1% [74.6%; 92.4%]; H = 2.68 [1.99; 3.62]

Test of heterogeneity:

Q d.f. p-value

50.37 7 < 0.0001

ASSESSMENT OF SMALL STUDIES BIAS

Linear regression test of funnel plot asymmetry

Test result: t = 1.16, df = 13, p-value = 0.2668

Egger’s method

Sample estimates:

bias se.bias intercept se.intercept

2.2565 1.9449 -0.4940 0.2576

Details:

- multiplicative residual heterogeneity variance (tau^2 = 23.9828)

- predictor: standard error

- weight: inverse variance

- reference: Egger et al. (1997), BMJ

Peters’ method

Test result: t = 1.39, df = 13, p-value = 0.1866

Sample estimates:

bias se.bias intercept se.intercept

6282927.9502 4506544.7168 -0.3578 0.1982

Details:

- multiplicative residual heterogeneity variance (tau^2 = 137.9385)

- predictor: inverse of total sample size

- weight: inverse variance of average event probability

- reference: Peters et al. (2006), JAMA


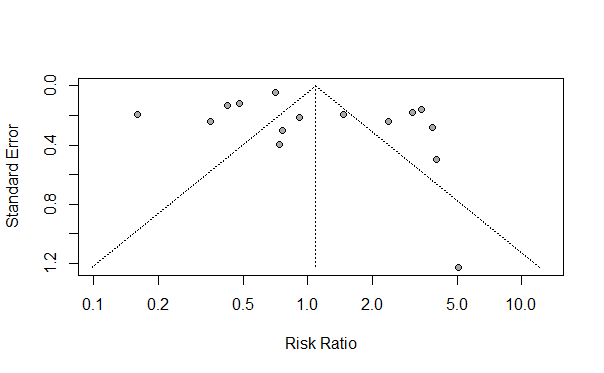


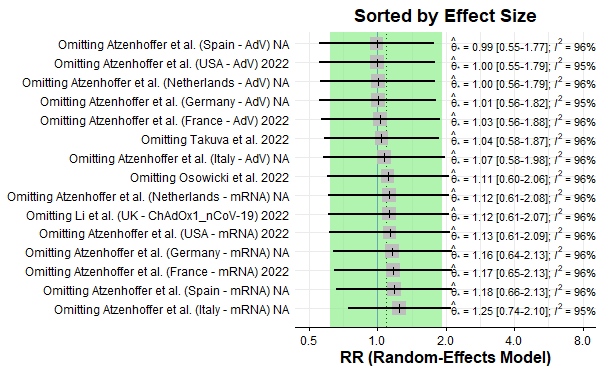

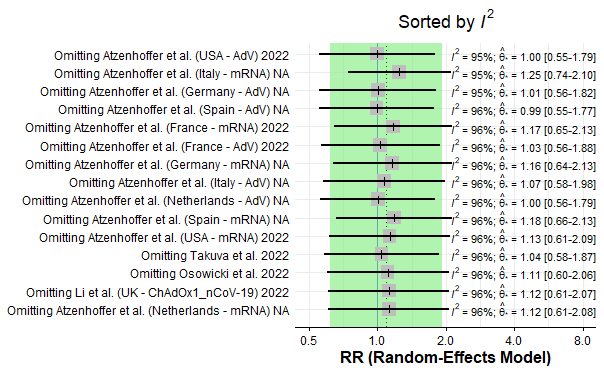


**GBS risk between observed and expected GBS cases for mRNA COVID-19 vaccines**Number of studies: k = 9

Number of observations: o = 2202162122

Number of events: e = 3853.57

RR 95%-CI z p-value

Random effects model 0.3238 [0.2253; 0.4654] -6.09 < 0.0001

Prediction interval [0.0896; 1.1709]

Quantifying heterogeneity:

tau^2 = 0.2612 [0.0887; 1.2109]; tau = 0.5111 [0.2978; 1.1004]

I^2 = 92.7% [88.2%; 95.4%]; H = 3.69 [2.92; 4.67]

Test of heterogeneity:

Q d.f. p-value

108.89 8 < 0.0001

Details on meta-analytical method:

- Inverse variance method

- Paule-Mandel estimator for tau^2

- Q-Profile method for confidence interval of tau^2 and tau

- Prediction interval based on t-distribution (df = 7)

No outliers detected (random-effects model).

**Funnel plot**


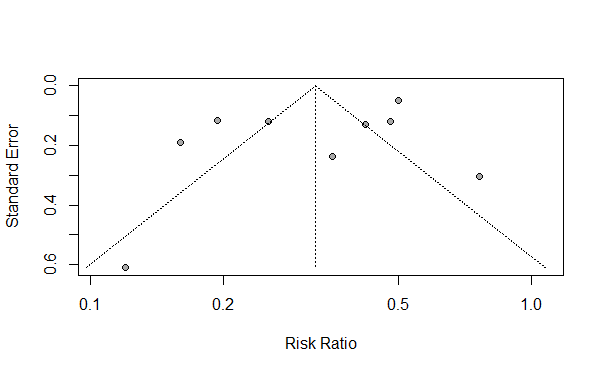


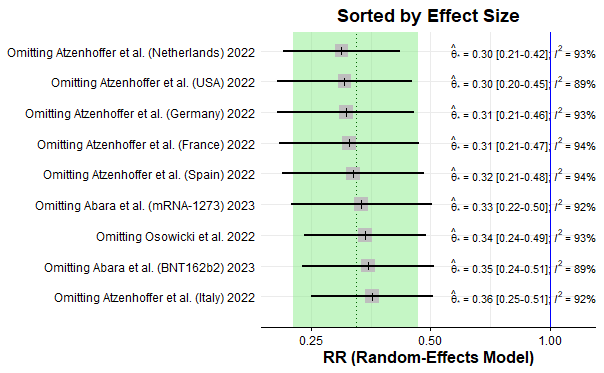

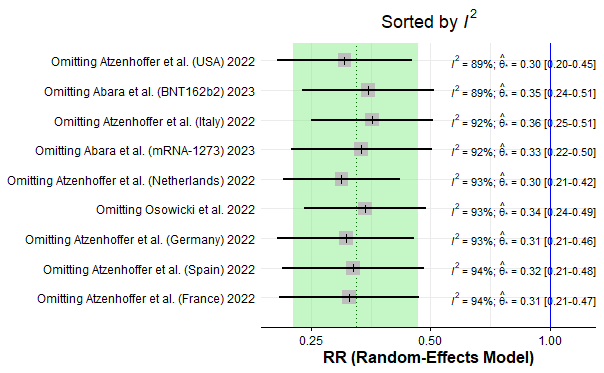


**Influence Analysis**

**GBS risk between observed and expected GBS cases for vector-based COVID-19 vaccines**

Number of studies: k = 10

Number of observations: o = 184886390

Number of events: e = 871.89

RR 95%-CI t p-value

Random effects model 2.3694 [1.6698; 3.3620] 5.58 0.0003

Prediction interval [0.9093; 6.1736]

Quantifying heterogeneity:

tau^2 = 0.1485 [0.0233; 0.8172]; tau = 0.3854 [0.1526; 0.9040]

I^2 = 65.7% [32.8%; 82.5%]; H = 1.71 [1.22; 2.39]

Test of heterogeneity:

Q d.f. p-value

26.23 9 0.0019

Details on meta-analytical method:

- Inverse variance method

- Paule-Mandel estimator for tau^2

- Q-Profile method for confidence interval of tau^2 and tau

- Hartung-Knapp adjustment for random effects model (df = 9)

- Prediction interval based on t-distribution (df = 8)

Identified outliers (random-effects model)

"Li et al. (UK) 2022"

Results with outliers removed

Number of studies: k = 9

Number of observations: o = 184886390

Number of events: e = 871.89

RR 95%-CI t p-value

Random effects model 2.5850 [1.9866; 3.3636] 8.32 < 0.0001

Prediction interval [1.4344; 4.6584]

Quantifying heterogeneity:

tau^2 = 0.0490 [0.0000; 0.3638]; tau = 0.2214 [0.0000; 0.6032]

I^2 = 51.8% [0.0%; 77.4%]; H = 1.44 [1.00; 2.10]

Test of heterogeneity:

Q d.f. p-value

16.59 8 0.0346

ASSESS FOR SMALL STUDIES BIAS

Linear regression test of funnel plot asymmetry

Eggers method

Test result: t = -0.40, df = 8, p-value = 0.6997

Sample estimates:

bias se.bias intercept se.intercept

-0.5882 1.4710 1.0388 0.3554

Details:

- multiplicative residual heterogeneity variance (tau^2 = 3.2148)

- predictor: standard error

- weight: inverse variance

- reference: Egger et al. (1997), BMJ

Peters methods

Test result: t = -0.06, df = 8, p-value = 0.9512

Sample estimates:

bias se.bias intercept se.intercept

-103379.9118 1635838.5901 0.9608 0.1599

Details:

- multiplicative residual heterogeneity variance (tau^2 = 15.2379)

- predictor: inverse of total sample size

- weight: inverse variance of average event probability

- reference: Peters et al. (2006), JAMA

Funnel plot


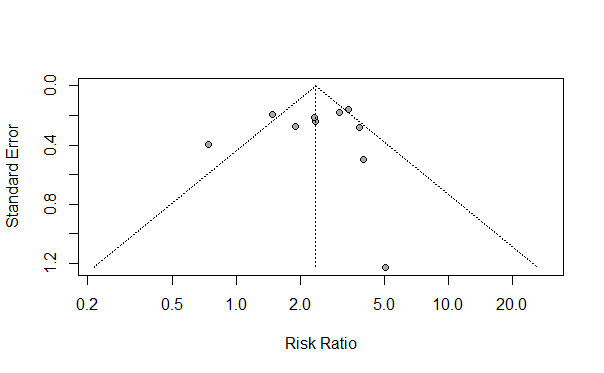


Influence analysis


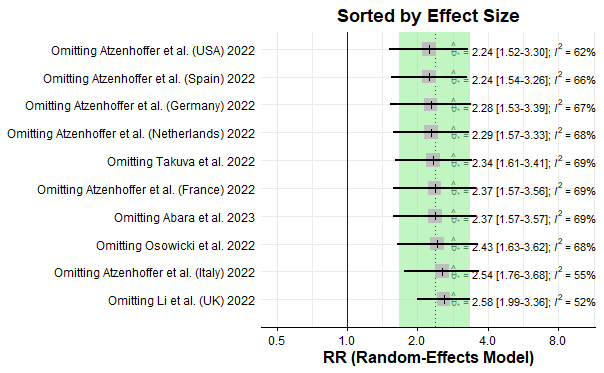

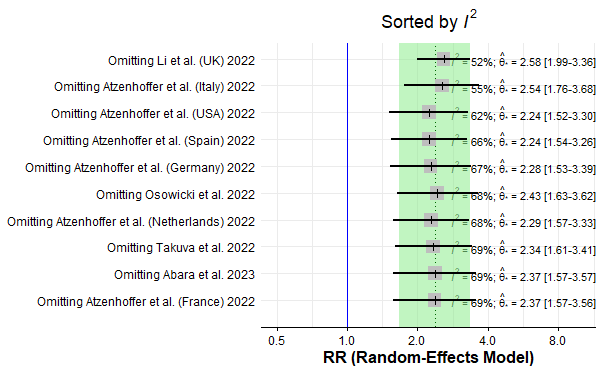

Supplement: Supplementary file 1 — Supplementary file1 (DOCX 344 KB) [file 415_2024_12186_MOESM1_ESM.docx]
